# Associations of ATP-Sensitive Potassium Channel’s Gene Polymorphisms With Type 2 Diabetes and Related Cardiovascular Phenotypes

**DOI:** 10.3389/fcvm.2022.816847

**Published:** 2022-03-23

**Authors:** Cheng Liu, Yanxian Lai, Tianwang Guan, Junfang Zhan, Jingxian Pei, Daihong Wu, Songsong Ying, Yan Shen

**Affiliations:** ^1^Department of Cardiology, Guangzhou First People’s Hospital, South China University of Technology, Guangzhou, China; ^2^Department of Cardiology, Guangzhou First People’s Hospital, Guangzhou Medical University, Guangzhou, China; ^3^Department of Health Management Center, Guangzhou First People’s Hospital, South China University of Technology, Guangzhou, China; ^4^Department of Cardiology, The Second Affiliated Hospital of Guangzhou Medical University, Guangzhou, China; ^5^Department of Gastroenterology, Guangzhou First People’s Hospital, South China University of Technology, Guangzhou, China

**Keywords:** type 2 diabetes, ATP-sensitive potassium channels, gene polymorphism, genotype, cardiovascular phenotype

## Abstract

Type 2 diabetes (T2D) is characterized by increased levels of blood glucose but is increasingly recognized as a heterogeneous disease, especially its multiple discrete cardiovascular phenotypes. Genetic variations play key roles in the heterogeneity of diabetic cardiovascular phenotypes. This study investigates possible associations of ATP-sensitive potassium channel (*KATP*) variants with cardiovascular phenotypes among the Chinese patients with T2D. Six hundred thirty-six patients with T2D and 634 non-diabetic individuals were analyzed in the study. Nine *KATP* variants were determined by MassARRAY. The *KATP rs2285676* (*AA* + *GA*, OR = 1.43, 95% CI: 1.13–1.81, *P* = 0.003), *rs1799858* (*CC*, OR = 1.42, 95% CI: 1.12–1.78, *P* = 0.004), and *rs141294036* (*CC*, OR = 1.45, 95% CI: 1.15–1.83, *P* = 0.002) are associated with increased T2D risk. A follow-up of at least 45.8-months (median) indicates further association between the 3 variants and risks of diabetic-related cardiovascular conditions. The associations are categorized as follows: new-onset/recurrent acute coronary syndrome (ACS) (*rs2285676/AA* + *GA*, HR = 1.37, 95% CI: 1.10–1.70, *P* = 0.005; *rs141294036/TT* + *CT*, HR = 1.59, 95% CI: 1.28–1.99, *P* < 0.001), new-onset stroke (*rs1799858/CC*, HR = 2.58, 95% CI: 1.22–5.43, *P* = 0.013; *rs141294036/CC*, HR = 2.30, 95% CI: 1.16–4.55, *P* = 0.017), new-onset of heart failure (HF) (*rs1799858/TT* + *CT*, HR = 2.78, 95% CI: 2.07–3.74, *P* < 0.001; *rs141294036/TT* + *CT*, HR = 1.45, 95% CI: 1.07–1.96, *P* = 0.015), and new-onset atrial fibrillation (AF) (*rs1799858/TT* + *CT*, HR = 2.05, 95% CI: 1.25–3.37, *P* = 0.004; *rs141294036/CC*, HR = 2.31, 95% CI: 1.40–3.82, *P* = 0.001). In particular, the *CC* genotype of *rs1799858* (OR = 2.38, 95% CI: 1.11–5.10, *P* = 0.025) and *rs141294036* (OR = 1.95, 95% CI: 1.04–3.66, *P* = 0.037) are only associated with the risk of ischemic stroke while its counterpart genotype (*TT* + *CT*) is associated with the risks of HF with preserved ejection fraction (HFpEF) (*rs1799858*, OR = 3.46, 95% CI: 2.31–5.18, *P* < 0.001) and HF with mildly reduced ejection fraction (HFmrEF) (*rs141294036*, OR = 2.74, 95% CI: 1.05–7.15, *P* = 0.039). Furthermore, the 3 variants are associated with increased risks of abnormal serum levels of triglyceride (TIRG) (≥ 1.70 mmol/L), low-density lipoprotein cholesterol (LDL-C) (≥ 1.40 mmol/L), apolipoprotein B (ApoB) (≥ 80 mg/dL), apolipoprotein A-I (ApoA-I) level (< 120 mg/dL), lipoprotein(a) Lp(a) (≥ 300 mg/dL) and high-sensitivity C-reactive protein (HsCRP) (≥ 3.0 mg/L) but exhibited heterogeneity (all *P* < 0.05). The *KATP rs2285676*, *rs1799858*, and *rs141294036* are associated with increased risks of T2D and its related cardiovascular phenotypes (ACS, stroke, HF, and AF), but show heterogeneity. The 3 *KATP* variants may be promising markers for diabetic cardiovascular events favoring “genotype-phenotype” oriented prevention and treatment strategies.

## Introduction

Type 2 diabetes (T2D) is characterized by increased levels of blood glucose owing to the combination of insulin resistance (IR), impaired insulin secretion, or (and) glucagon excess (1). The T2D is a serious public health hazard worldwide, especially in the present day China. Cardiovascular diseases (CVDs), including arteriosclerosis cardiovascular disease (ASCVD), heart failure (HF), and atrial fibrillation (AF), are the prominent causes of death and disability in patients with T2D. T2D and its related CVDs result in a complex phenotype profile that is caused by many individual genetic events and environmental factors (2). Besides the environmental factors, evidences indicate that genetic variants may play a pivotal role in the initiation and progression of diabetic cardiovascular phenotypes. The complex genetic background poses more challenges in the prevention and treatment of T2D and its related CVDs. Therefore, early assessments of populations at greater genetic predisposition for different cardiovascular phenotypes are important and necessary for early and comprehensive management of T2D and its associated comorbidities.

Adenosine Triphosphate-sensitive potassium channels (*KATP*) are important mediators as they couple high-fidelity metabolic sensors (e.g., glucose homeostasis) to end effectors for cardiovascular protection (e.g., ASCVD, HF, and AF) (3). Their key function makes them a novel promising target to improve diabetes and its cardiovascular complications. The *KATP* is a protein complex with four Kir6.x subunits (inwardly-rectifying potassium channel, Kir6.1 and Kir6.2) and four SURx subunits (sulfonylurea receptor, SUR1 and SUR2). Different combinations of the two subunits and different isoforms produce channels with diverse physiological and pathological properties, exhibiting a high degree of genetic polymorphism.

As vital high-fidelity metabolic sensors, *KATP* single nucleotide polymorphisms (SNPs) are relevant to T2D risk in the global population (4, 5), but the relationship presents characteristics with genetic heterogeneity in different regions and races of the world. In Caucasian populations, the *KATP* variants are associated with increased T2D risk in the Europeans (e.g., *rs5219*) (4), the French (e.g., *rs1799859*) (6), the Turks (e.g., *rs1799854* and *rs1799859*) (7), and the Iranians (e.g., *rs757110*) (8). In contrast, the *KATP rs757110* is associated with decreased T2D risk in the British (9). However, some *KATP* variants are not associated with T2D risk, such as *rs1799845* and *rs1801261* in the British (10), *rs5219* in the Arabs (11), *rs5219* and *rs757110* in the Siberians (5). In non-Caucasian populations, the *KATP* variants are associated with increased T2D risk in the Japanese (e.g., *rs5219* and *rs757110*) (12), the Mongolians (e.g., rs1799858 and *rs2074308*) (13), the Indians (e.g., *chr11:17417205C* > *T* and *rs5210*) (14) and the Nigerians (e.g., *rs1799854*) (15) rather than the Punjab population of India (e.g., *rs1799854* and *rs1801261*) (16) and the Sub-Saharan Africans (e.g., *rs5219* in the Ghanaians and the Nigerians) (17). Previous meta-analysis shows that *KATP rs5219* is a common variant for T2D risk in the world population, and has similar effect on the susceptibility risk to T2D in the Europeans and the East Asian (e.g., the Japanese and the Koreans), but the loci are not associated with T2D risk in the Chinese Han population (4).

As an important end effector of cardiac protection, only few studies report the relationships between *KATP* mutations and cardio-cerebrovascular disease risk. The *KATP* variants are associated with T2D-related stroke in the Poles (e.g., *rs1799854*) (18), coronary atherosclerotic heart disease (CAD) in the British (e.g., *rs757110* and *rs61688134*) (9, 19), HF in the Ukrainians (e.g., *rs5210*, *rs5219* and *rs757110*) (20) and the Americans (e.g., *rs5219*) (21), and AF in the Americans (e.g., *rs72554071*) (22). Importantly, these cardiovascular risks may be higher under the crosstalk between *KATP* mutation (e.g., *rs757110*) and traditional risk factors (e.g., smoking) (23).

These findings suggest an ethnic-specific/geographical genetic pleiotropy of *KATP* variants manifested as different clinical phenotypes of diabetes. However, the genotype-phenotype correlation of *KATP* mutations on T2D and its related CVDs in the Chinese population are rarely reported. This study characterizes the genotype-phenotype correlation between *KATP* variants and T2D/associated CVDs in China.

## Materials and Methods

### Study Design and Participants

A total of 1,270 participants from South China enrolled in this study, starting on January 1, 2013 and ending on December 31, 2020, were divided into two groups: non-T2D group (*N* = 634) and T2D group (*N* = 636). The inclusion criteria for the case group were as follows: (1) individuals aged 18 years or older who were newly diagnosed with T2D referring to the 2012 clinical practice guidelines and its update of the American Diabetic Association (24, 25); (2) patients with T2D who had not received therapy; and (3) patients who signed the consent form. Inclusion criteria for the control group were as follows: (1) individuals aged 18 years or older, (2) in healthy conditions based on anamnesis and physical examination results, (3) no evidence of T2D or family history of T2D, and (4) patients who signed the consent form. The non-T2D participants, got a health examination at the same hospital during the same period, and were enrolled as the control group. Subjects were excluded from enrollment in the study upon presenting (1) a history of hypertension (HTN), CAD, HF, and stroke. However, the history of lacunar cerebral infarcts (LCIs) or (and) atrial fibrillation (AF) at enrollment, the new onset of HTN, CAD, HF, and stroke during the follow-up will be not considered as exclusion criteria. The subjects who had a history of LCI or/and AF but no history of HTN, CAD, HF, and stroke at the time of enrollment were not considered as exclusion criteria. Stroke and LCI were evaluated by magnetic resonance imaging (MRI) or (and) computed tomography (CT). Stroke was evaluated at the time of enrollment by MRI or (and) CT; (2) high levels of aspartate aminotransferase (AST) and alanine transaminase (ALT) (more than 3 times the upper limit of the normal); (3) low estimated glomerular filtration rate (less than 90 ml/min•1.73 m^2^); (4) or (and) any other factors that contribute to serum lipid abnormalities, such as medical disorders (thyroid disease, Cushing’s syndrome, and pheochromocytoma) and drugs [thiazide diuretics, beta-receptor blockers (BBs), steroid, estrogen, progesterone, immunosuppressants, or retinoids]. Dyslipidemia is defined as an increase in triglyceride levels (TIRG) ≥ 1.7 mmol/L, total cholesterol (TC) ≥ 4.0 mmol/L, low-density lipoprotein cholesterol (LDL-C) ≥ 1.4 mmol/L, high-density lipoprotein cholesterol (HDL-C) < 1.0 mmol/L, apolipoprotein B (ApoB) ≥ 80 mg/dL, apolipoprotein A-I (ApoA-I) < 120 mg/dL or lipoprotein (a) [Lp(a) ≥ 300 mg/dL], and any their combinations. All patients with dyslipidemia were newly diagnosed at the time of enrollment in this study. Dyslipidemia diagnosis is in accordance with the recommendations on lipid disorders management of ESC/EAS (26). Echocardiography is evaluated using the standard sections at enrollment according to the recommendations of the American Society of Echocardiography (26). Blood biochemistry analyses were carried out at the time of enrollment according to standard analytical methods.

### Endpoints

In this study, the primary endpoints are defined as the risks of new-onset/recurrent acute coronary syndrome (ACS), new-onset of stroke, new-onset of HF, and new-onset of AF during the follow-up. The secondary endpoints are defined as the risks of LCI at enrollment, AF at enrollment, and the total AF risk at the end of the follow-up period. These endpoint events were identified from three information sources, including the Center for Disease Control and Prevention, medical records, and participants and their families. Subjects were enrolled in the study on their first day of T2D diagnosis. December 31, 2020 was the final follow-up date. During the follow-up, events were diagnosed with reference to the following guidelines: (1) new-onset/recurrent ACS is defined as ST-elevation of myocardial infarction (MI), non-ST-elevation of MI or unstable angina, confirmed by cardiac biomarkers and coronary angiography. (2) LCIs are small infarcts (2–20 mm in diameter) caused by the occlusion of small penetrating end-arteries. New-onset of stroke is defined as the presence of a focal/global cerebral event including hemorrhagic stroke (HS), ischemic stroke (IS), and cardiogenic stroke (CS). Both LCI and strokes were evaluated by MRI or (and) CT at enrollment. (3) New-onset of HF is defined as the first occurrence of a HF admission (including at least one overnight hospitalization to emergency) (27), including HF with reduced ejection fraction [HFrEF, left ventricular ejection fraction (LVEF) ≤ 40%], HF with mildly reduced ejection fraction (HFmrEF, LVEF = 41–49%), and HF with preserved ejection fraction (HFpEF, LVEF ≥ 50%). (4) The AF is assessed according to prior medical records or electrocardiogram records from the enrollment to follow-up, including AF at enrollment and new-onset of AF. The new-onset of AF is defined as the newly detected AF in participants with no previous diagnosis of this condition before enrollment.

### Genotyping of ATP-Sensitive Potassium Channels Single Nucleotide Polymorphisms

Nine *KATP* SNPs (*rs2285676*, *rs11046182*, *rs1799858*, *rs4148671*, *rs78148713*, *rs145456027*, *rs147265929*, *rs61928479*, and *rs141294036*) were identified using the MassARRAY (Sequenom) system as previously described (26). Specific primers for the 9 *KATP* SNPs are shown in Supplementary Table 1. The 9 *KATP* SNPs have 100% accurate genotypes for further analyses.

### Statistical Analysis

All analyses were performed using PASS 15.0 (Statistical Solution Ltd, Cork, Ireland) and SPSS 20.0 (SPSS, Chicago, IL). LDlink (LDmatrix) online tool^1^ was used to calculate the linkage disequilibrium (LD) for the 9 KATP variants in the Chinese populations as shown in Supplementary Figure 1. The Hardy-Weinberg equilibrium was investigated for both non-diabetic and diabetic subjects as shown in Supplementary Table 2. Data of categorical variables are presented as numbers (percentages) while continuous variables are presented as mean ± standard deviation. The difference among the subjects without or with T2D was analyzed by χ^2^-test for the categorical variables, or by independent sample *t*-test for the continuous variables. The dominant models for the minor allele of the 9 *KATP* SNPs were tested to obtain odds ratios (ORs)/hazard ratios (HRs), 95% confidence intervals (CIs), and *P*-values. Associations of the 9 *KATP* SNPs with T2D were assessed using binary logistic regression model as well as LCI, different types of dyslipidemia, and low-grade inflammation levels [high-sensitivity C-reactive protein (HsCRP) ≥ 3.0 mg/L]. Associations of the 9 *KATP* SNPs with new-onset/recurrent ACS were assessed using Cox proportional hazards regression model; the new-onset of stroke, HF, and AF were assessed similar to ACS. In particular, the relationships between *KATP* variants and different stroke subtypes (HS, IS and CS) were assessed using multinomial logistic regression model; the different HF subtypes (HFpEF, HFmrEF, and HFrEF) were assessed similarly. Bonferroni correction was conducted to protect from Type I error. Further, the heritability (explained variance,%) of *KATP* variants for events was calculated according to the method reported by Yang et al. (28). All probabilities are two-tailed. A *P*-value < 0.05 is considered statistically significant.

## Results

### Characteristics of the Study Subjects

Among all the study subjects (*N* = 1,270), 4.1% were presented with a history of LCI (52 patients), and 3.5% with a history of AF (44 patients) at the time of enrollment, as shown in Table 1. Subjects with or without T2D at enrollment indicate obvious differences in LCI incidence rates (*P* = 0.024), levels of systolic blood pressure (SBP, *P* < 0.001) and diastolic blood pressure (DBP, *P* < 0.001), white blood cell count (WBC, *P* < 0.001), serum levels of TRIG (*P* < 0.001), HDL-C (*P* < 0.001), ApoA-I (*P* = 0.018), uric acid (UA, *P* < 0.001), HsCRP (*P* = 0.030), left atrial end-diastolic dimension (LAD, *P* = 0.012), LVEF (*P* = 0.028), left ventricular mass index (LVMI, *P* = 0.001) and E/e’ ratio (*P* = 0.003). During the follow-up period, none of the study subjects in the control group progressed to T2D, none dropped out of the study, and the subjects suffered from cardiovascular conditions are as follows: new-onset/recurrent ACS (416 patients, 32.8%), new-onset of stroke (58 patients, 4.7%), new-onset of HF (282 patients, 22.2%) and new-onset of AF (94 patients, 7.4%), as shown in Supplementary Table 3. Subjects with or without T2D present obvious differences for new-onset of HF (*P* = 0.005) and HF subtypes (*P* = 0.013); the differences remain significant upon factoring the medications in the analyses, such as mineralocorticoid receptor antagonist (MRA, *P* = 0.047), BBs (*P* = 0.015), and renin-angiotensin system inhibitors (RSIs, *P* < 0.001) at the end of follow-up.

**TABLE 1:** Baseline characteristics of the study participants.

	Non-T2D	T2D	*P-*value
Sample (N)	634	636	–
Male:Female (N)	474: 160	466: 170	0.544
Age (Y)	63.6 ± 10.9	64.6 ± 10.9	0.107
Smoking (N/%)	332 (52.4)	316 (49.7)	0.339
Alcohol consumption (N/%)	96 (15.1)	84 (13.2)	0.332
**SBP (mmHg)**	**119.4 ± 10.3**	**123.3 ± 8.6**	**<0.001**
**DBP (mmHg)**	**69.1 ± 6.4**	**72.7 ± 7.4**	**<0.001**
HR(bpm)	74 ± 11	75 ± 12	0.368
BMI (kg/m^2^)	24.4 ± 4.0	25.0 ± 4.2	0.009
**Medical condition**			
**LCI (N/%)**	**18 (2.8)**	**34 (5.3)**	**0.024**
AF (N/%)	22 (3.5)	22 (3.5)	0.992
**Blood biochemical index**			
**WBC (× 10^9^/L)**	**7.45 ± 1.70**	**8.04 ± 2.19**	**<0.001**
HGB (g/L)	132.8 ± 17.9	131.0 ± 17.6	0.076
PLT (× 10^9^/L)	231.3 ± 59.0	234.2 ± 68.8	0.427
**TRIG (mmol/L)**	**1.42 ± 0.77**	**1.80 ± 1.12**	**<0.001**
CHOL (mmol/L)	4.41 ± 1.24	4.41 ± 1.21	0.910
**HDL-C (mmol/L)**	**1.16 ± 0.33**	**1.09 ± 0.28**	**<0.001**
LDL-C (mmol/L)	2.44 ± 0.86	2.37 ± 0.84	0.138
**ApoA-I (mg/mL)**	**106.8 ± 24.7**	**103.4 ± 23.1**	**0.018**
ApoB (mg/mL)	91.8 ± 31.6	92.8 ± 34.5	0.591
Lp-A(mg/dL)	294.5 ± 308.0	286.8 ± 289.0	0.647
**FBG (mmol/L)**	**5.16 ± 0.70**	**6.53 ± 1.54**	**<0.001**
**P2hBS (mmol/L)**	**6.94 ± 1.49**	**9.61 ± 2.81**	**<0.001**
**HbA1C (%)**	**5.5 ± 0.7**	**6.6 ± 1.5**	**<0.001**
Scr (μmol/L)	72.9 ± 13.7	72.2 ± 12.4	0.287
BUN (mmol/L)	5.46 ± 1.80	5.55 ± 1.61	0.366
**UA (μ mol/L)**	**343.7 ± 73.8**	**373.6 ± 81.4**	**<0.001**
ALT (U/L)	24.2 ± 14.0	23.4 ± 13.6	0.277
AST (U/L)	25.8 ± 16.5	25.9 ± 16.3	0.875
Alb (g/L)	37.6 ± 4.4	37.6 ± 3.7	0.738
Na^+^ (mmol/L)	140.4 ± 3.2	140.3 ± 3.3	0.440
K^+^ (mmol/L)	3.73 ± 0.42	3.73 ± 0.39	0.843
**HsCRP (mg/L)**	**10.9 ± 20.0**	**13.3 ± 20.3**	**0.030**
ACE (U/L)	35.1 ± 21.6	33.0 ± 19.7	0.065
Renin (pg/mL)	24.8 ± 26.0	25.8 ± 29.9	0.544
Ang I (ng/L)	2.10 ± 1.57	2.34 ± 1.71	0.128
Ang II (ng/L)	66.4 ± 92.7	66.3 ± 95.6	0.971
ALD (ng/L)	187.0 ± 124.2	177.7 ± 120.4	0.175
**Echocardiography**			
RAD (mm)	33.4 ± 3.4	33.5 ± 2.7	0.687
RVD (mm)	17.4 ± 1.8	17.4 ± 1.8	0.693
**LAD (mm)**	**30.8 ± 5.3**	**31.5 ± 5.5**	**0.012**
LVD (mm)	47.8 ± 5.6	48.2 ± 5.5	0.167
**LVEF (%)**	**55.6 ± 9.1**	**54.5 ± 9.9**	**0.028**
**LVMI (g/m^2^)**	**102.6 ± 29.7**	**108.4 ± 32.8**	**0.001**
**E/e’ ratio**	**10.6 ± 3.8**	**11.3 ± 4.3**	**0.003**

*The bold values mean P value < 0.05.*

### Association Between ATP-Sensitive Potassium Channels Single Nucleotide Polymorphisms and Type 2 Diabetes Risk

**FIGURE 1:**
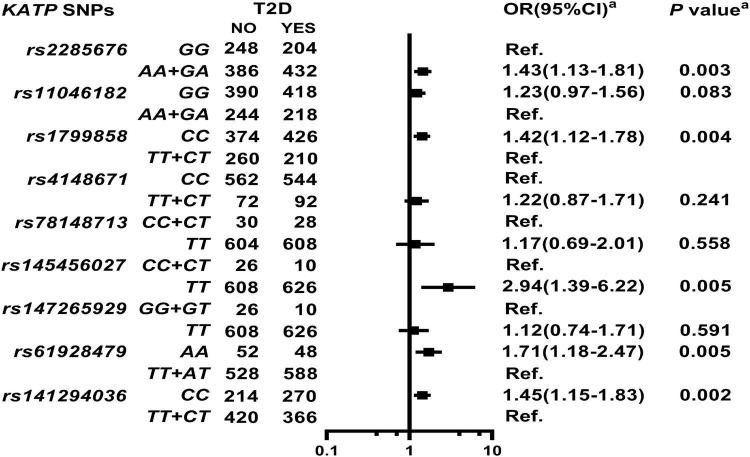
Association of ATP-sensitive potassium channels (*KATP*) single nucleotide polymorphisms (SNPs) with Type 2 diabetes (T2D) in the study participants. *^a^*Model: After adjustment for gender, age, smoking, alcohol consumption, body mass index (BMI), systolic blood pressure (SBP), diastolic blood pressure (DBP), high-sensitivity C-reactive protein (HsCRP), and renin-angiotensin-aldosterone system (RAAS activity) [angiotensin converting enzyme (ACE), renin, angiotensin I (Ang I), angiotensin II (Ang II), and aldosterone (ALD)].

As shown in Figure 1 and Supplementary Table 4, *KATP* SNPs *rs2285676* (*AA* + *GA*, adjusted OR = 1.43, 95% CI: 1.13–1.81, *P* = 0.003), *rs1799858* (*CC*, adjusted OR = 1.42, 95% CI: 1.12–1.78, *P* = 0.004), *rs145456027* (*TT*, adjusted OR = 2.94, 95% CI: 1.39–6.22, *P* = 0.005), *rs61928479* (*AA*, adjusted OR = 1.71 95% CI: 1.18–2.47, *P* = 0.005), and *rs141294036* (*CC*, adjusted OR = 1.45, 95% CI: 1.15–1.83, *P* = 0.002) significantly correlate with moderate to high risk T2D except for *rs11046182*, *rs4148671*, *rs78148713*, and *rs147265929* (all adjusted *P* > 0.05).

### Association Between ATP-Sensitive Potassium Channels Single Nucleotide Polymorphisms and New-Onset/Recurrent Acute Coronary Syndrome Risk

**FIGURE 2:**
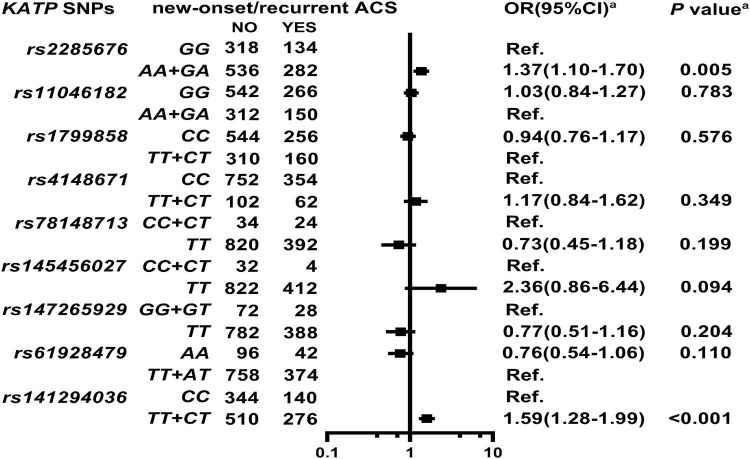
Association of *KATP* SNPs with new onset/recurrent ACS in the study participants. *^a^*Model: After adjustment for gender, age, smoking, alcohol consumption, BMI, white blood cell count (WBC), blood glucose levels [fasting blood sugar (FBS), postprandial blood glucose 2 h (P2hBS) and glycosylated hemoglobin (HbA1C), liver function [alanine aminotransferase (ALT), aspartate aminotransferase (AST), and albumin (Alb)], renal function [serum creatinine (Scr), blood urea nitrogen (BUN) and uric acid (UA)], serum sodium and potassium levels, HsCRP, HbA1C, RAAS activity (ACE, renin, Ang I, Ang II, and ALD), dyslipidemia {triglyceride (TRIG), total cholesterol (TC), low-density lipoprotein cholesterol (LDL-C), apolipoproteinB (ApoB), high-density lipoprotein cholesterol (HDL-C), apolipoproteinA-I (ApoA-I), and lipoprotein(a) [Lp(a)]}, medical condition [type 2 diabetes (T2D), EH, heart failure (HF) and atrial fibrillation (AF)], NYHA functional classification, and echocardiography index [right ventricular end-diastolic diameter (RVD), right atrial end-diastolic dimension (RAD), left ventricular end-diastolic diameter (LVD), left atrial end-diastolic dimension (LAD), left ventricular mass index (LVMI), and left ventricular ejection fraction (LVEF)], and combined medication [antiplatelet drugs, warfarin, statins, renin-angiotensin system inhibitors (RSIs), beta-receptor blockers (BBs), mineralocorticoid receptor antagonist (MRA), calcium-channel blockers (CCBs), diuretics, digoxin, nitrates, and hypoglycemic agents].

As shown in Figure 2, Supplementary Table 5, and Supplementary Figure 2, *KATP rs2285676* (*AA* + *GA*, adjusted HR = 1.37, 95% CI: 1.10–1.70, *P* = 0.005) and *rs141294036* (*TT* + *CT*, adjusted HR = 1.59, 95% CI: 1.28–1.99, *P* < 0.001) are associated with a moderate risk of new-onset/recurrent ACS after a median follow-up of 45.8-months (IQR: 27.4–55.3 months).

### Association Between ATP-Sensitive Potassium Channels Single Nucleotide Polymorphisms and Risks of New-Onset Stroke

**FIGURE 3:**
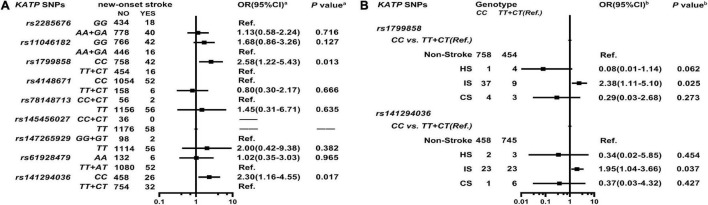
Association of *KATP* SNPs with the new-onset of stroke in the study participants. *^a^*Model **(A)** After adjustment for gender, age, smoking, alcohol consumption, BMI, WBC, blood glucose levels (FBS, P2hBS and HbA1C), liver function (ALT, AST and Alb), renal function (Scr, BUN and UA), serum sodium and potassium levels, HsCRP, RAAS activity (ACE, renin, Ang I, Ang II, and ALD), dyslipidemia [TRIG, TC, LDL-C, ApoB, HDL-C, ApoA-I and Lp(a)], medical condition [T2D, EH, CAD(ACS), AF, and LCI], NYHA functional classification, and echocardiography index (RVD, RAD, LVD, LAD, and LVEF), and combined medication (antiplatelet drugs, warfarin, statins, RSIs, BBs, MRA, CCBs, diuretics, digoxin, nitrates, and hypoglycemic agents). *^b^*Model **(B)** It is the same as Model **(A)**.

As shown in Figure 3A, Supplementary Table 6, and Supplementary Figure 3, *KATP rs1799858* (*CC*, adjusted HR = 2.58, 95% CI: 1.22–5.43, *P* = 0.013) and *rs141294036* (*CC*, adjusted HR = 2.30, 95% CI: 1.16–4.55, *P* = 0.017) correlate with increased risk of new-onset stroke based on the median follow-up period of 50.8-months (IQR: 44.0–58.7 months). As shown in Figure 3B and Supplementary Table 7, further analyses of stroke subtype indicate that *rs1799858* (*CC*, adjusted OR = 2.38, 95% CI: 1.11–5.10, *P* = 0.025) and *rs141294036* (*CC*, adjusted OR = 1.95, 95% CI: 1.04–3.66, *P* = 0.037) are associated only with IS.

### Association Between ATP-Sensitive Potassium Channels Single Nucleotide Polymorphisms and Heart Failure Risk

**FIGURE 4:**
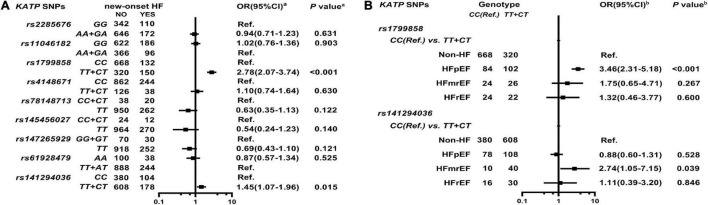
Association of *KATP* SNPs with incidence of HF in the study participants. *^a^*Model **(A)** After adjustment for gender, age, smoking, alcohol consumption, BMI, WBC, blood glucose levels (FBS, P2hBS, and HbA1C), liver function (ALT, AST, and Alb), renal function (Scr, BUN, and UA), serum sodium and potassium levels, HsCRP, RAAS activity (ACE, renin, Ang I, Ang II, and ALD), dyslipidemia [TRIG, TC, LDL-C, ApoB, HDL-C, ApoA-I, and Lp(a)], medical condition [T2D, HTN, CAD(ACS), and AF], echocardiography index (RVD, RAD, LVD, LAD, and LVEF), and combined medication (antiplatelet drugs, warfarin, statins, RSIs, BBs, MRA, CCBs, diuretics, digoxin, nitrates, and hypoglycemic agents). *^b^*Model **(B)** It is the same as Model **(A)**.

As shown in Figure 4A, Supplementary Table 8, and Supplementary Figure 4, *KATP rs1799858* (*TT* + *CT*, adjusted HR = 2.78, 95% CI: 2.07–3.74, *P* < 0.001) and *rs141294036* (*TT* + *CT*, adjusted HR = 1.45, 95% CI: 1.07–1.96, *P* = 0.015) are associated with high HF risk based on a median follow-up of 48.1-months (IQR: 39.1–56.8 months). As shown in Figure 4B and Supplementary Table 9, further analyses of HF subtypes show that *rs1799858* is associated only with high HFpEF risk (*TT* + *CT*, adjusted OR = 3.46, 95% CI: 2.31–5.18, *P* < 0.001) while *rs141294036 is* associated with high HFmrEF risk (*TT* + *CT*, adjusted OR = 2.74, 95% CI: 1.05–7.15, *P* = 0.039).

### Association Between ATP-Sensitive Potassium Channels Single Nucleotide Polymorphisms and Atrial Fibrillation Risk

**FIGURE 5:**
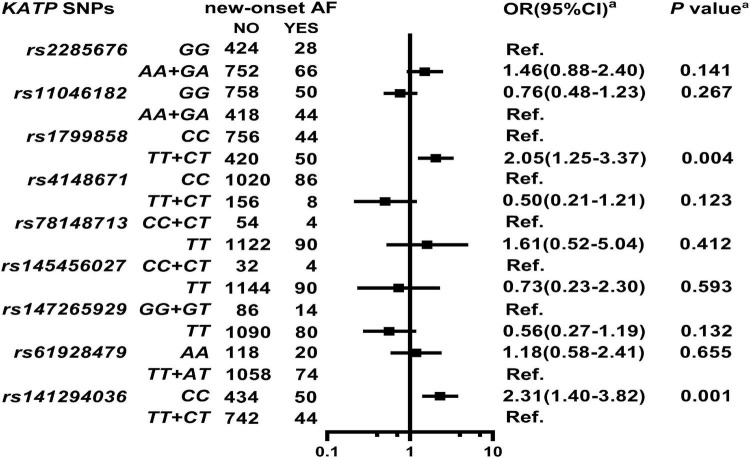
Association of *KATP* SNPs with the new-onset of AF in the study participants. *^a^*Model: After adjustment for gender, age, smoking, alcohol consumption, BMI, WBC, blood glucose levels (FBS, P2hBS and HbA1C), liver function (ALT, AST and Alb), renal function (Scr, BUN, and UA), serum sodium and potassium levels, HsCRP, RAAS activity (ACE, renin, Ang I, Ang II and ALD), dyslipidemia [TRIG, TC, LDL-C, ApoB, HDL-C, ApoA-I and Lp(a)], medical condition [T2D, HTN, CAD(ACS), and HF], echocardiography index (RVD, RAD, LVD, LAD, and LVEF), and combined medication (antiplatelet drugs, warfarin, statins, RSIs, BBs, MRA, CCBs, diuretics, digoxin, nitrates, and hypoglycemic agents).

As shown in Supplementary Table 10, two *KATP* variants are associated with high AF risk at enrollment as follows: *rs1799858* (*TT* + *CT*, adjusted OR = 2.15, 95% CI: 1.06–4.39, *P* = 0.035) and *rs141294036* (*CC*, adjusted OR = 2.42, 95% CI: 1.15–5.07, *P* = 0.020). As shown in Figure 5, Supplementary Table 11, and Supplementary Figure 5, *rs1799858* (*TT* + *CT*, adjusted HR = 2.05, 95% CI: 1.25–3.37, *P* = 0.004) and *rs141294036* (*CC*, adjusted HR = 2.31, 95% CI: 1.40–3.82, *P* = 0.001) are associated with high risk of new-onset of AF based on a median follow-up of 50.1-months (IQR: 42.7–58.4 months).

### Association Between ATP-Sensitive Potassium Channels Single Nucleotide Polymorphisms and Dyslipidemia/Inflammation Levels

**FIGURE 6:**
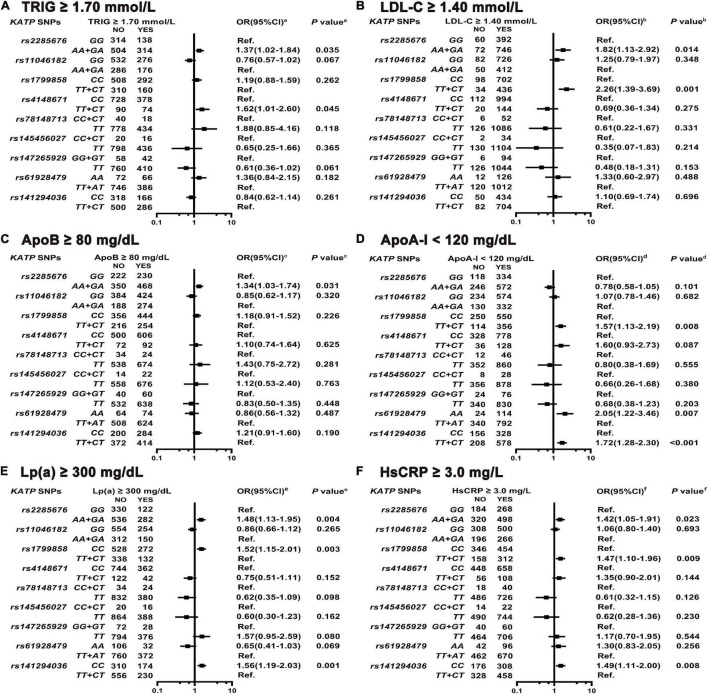
Association of *KATP* SNPs with abnormal serum levels of blood lipid and HsCRP in the study participants. *^a^*Model **(A)** After adjustment for gender, age, smoking, alcohol consumption, BMI, WBC, T2D, SBP, DBP, blood glucose levels (FBS, P2hBS, and HbA1C), liver function (ALT, AST, and Alb), renal function (Scr, BUN, and UA), serum sodium and potassium levels, HsCRP, RAAS activity (ACE, renin, Ang I, Ang II, and ALD), and dyslipidemia [TC, LDL-C, ApoB, HDL-C, ApoA-I, and Lp(a)]. *^b^*Model **(B)** Is the same as Model 6a except LDL-C, and including TRIG. *^c^*Model **(C)** Is the same as Model 6a except ApoB, and including TRIG. *^d^*Model **(D)** Is the same as Model 6a except ApoA-I, and including TRIG. *^e^*Model **(E)** Is the same as Model 6a except Lp(a), and including TRIG. *^f^*Model **(F)** After adjustment for gender, age, smoking, alcohol consumption, WBC, BMI, T2D, SBP, DBP, blood glucose levels (FBS, P2hBS, and HbA1C), liver function (ALT, AST, and Alb), renal function (Scr, BUN and UA), serum sodium and potassium levels, HsCRP, dyslipidemia (TRIG, TC, LDL-C, HDL-C, ApoA-I, and ApoB) and RAAS activity (ACE, renin, Ang I, Ang II, and ALD).

As shown in Figures 6A–E and Supplementary Tables 14–18, *KATP rs2285676* (*AA* + *GA* genotype) is associated with the elevated risk of higher TRIG serum levels (adjusted OR = 1.37, 95% CI: 1.02–1.84, *P* = 0.035), LDL-C (adjusted OR = 1.82, 95% CI: 1.13–2.92, *P* = 0.014), ApoB (adjusted OR = 1.34, 95% CI: 1.03–1.74, *P* = 0.031), and Lp(a) (adjusted OR = 1.48, 95% CI: 1.13–1.95, *P* = 0.004). The *rs1799858* (*TT* + *CT*) is associated with a higher risk of elevated LDL-C serum levels (adjusted OR = 2.26, 95% CI: 1.39–3.69, *P* = 0.001) and lower ApoA-I serum levels (adjusted OR = 1.57, 95% CI: 1.13–2.19, *P* = 0.008) while *CC* genotype is associated with higher Lp(a) serum levels (adjusted OR = 1.52, 95% CI: 1.15–2.01, *P* = 0.003). Similarly, *rs141294036* (*TT* + *CT*) is associated with the elevated risk of lower serum ApoA-I levels (adjusted OR = 1.72, 95% CI: 1.28–2.30, *P* < 0.001) while *CC* genotype is associated with higher Lp(a) serum levels (adjusted OR = 1.56, 95% CI: 1.19–2.03, *P* = 0.001). *KATP rs4148671* (*TT* + *CT*) only correlates with increased risk of higher serum TRIG levels (adjusted OR = 1.62, 95% CI: 1.01–2.60, *P* = 0.045) while *rs61928479* (*AA*) correlates with the elevated risk of lower serum ApoA-I levels only (adjusted OR = 2.05, 95% CI: 1.22–3.46, *P* = 0.007). Three *KATP* SNPs (*rs11046182*, *rs78148713* and *rs145456027*) do not associate with any type of lipid disorder [HDL-C, ApoB, ApoA-I, and Lp(a), all *P* > 0.05]. As shown in Figure 6F and Supplementary Table 21, *KATP rs1799858* (*TT* + *CT*, adjusted OR = 1.47, 95% CI: 1.10–1.96, *P* = 0.009), *rs2285676* (*AA* + *GA*, adjusted OR = 1.42, 95% CI: 1.05–1.91, *P* = 0.023), and *rs141294036* (*CC*, adjusted OR = 1.49, 95% CI: 1.11–2.00, *P* = 0.008) correlate with the increased risk of higher serum HsCRP levels.

## Discussion

### ATP-Sensitive Potassium Channels Variants and Type 2 Diabetes Risk

This study demonstrates that the Chinese participants with the genotypes of *rs2285676* (*AA* + *GA*), *rs1799858* (*CC*), and *rs141294036* (*TT* + *CT*) have a 42–45% increased risk of T2D, and the total heritability of the 3 *KATP* variants for T2D risk is 2.1% (Supplementary Table 4). These findings are in controversy with the previous studies by Baier et al. (14) who reported no association between *KATP rs2285676* and T2D in specific Amerindian-derived population, and by Odgerel et al. (13) who reported an association between the T-allele of *rs1799858* and T2D in diabetic Mongolians rather than the *CC* genotype. The subjects with high diabetic-risk genotypes with different *KATP* variants correlate with increased T2D risk, but the effects show the characteristics of ethnic heterogeneity as follows: The French (*rs1799859/AA* + *AG*, by 69%) (6), the Turks (*rs1799859/AA* + *AG*, by 3.8-fold) (7), the Finns (*rs1799859/AA* + *AG*, by 2-fold), the Japanese (*rs5219/KK* + *EK*, by 32%) (12), the Mongolians (*TT* + *CT* of *rs1799858* and *rs2074308*, approximately by 80%) (13) and the Pima Indians (*chr11:17417205/T-allele*, by 1-fold) (14). Two meta-analysis studies confirm that *KATP* mutations correlate with increased T2D risk (about by 15–16%) in both the Caucasians (e.g., the Europeans) and the non-Caucasians (e.g., the Japanese) (4, 5). Furthermore, *KATP* variants are associated with antidiabetic efficacy of sulfonylurea hypoglycemic agents (e.g., gliclazide) as well as non-sulfonylureas (e.g., repaglinide), especially secondary sulfonylurea failure (29). This phenomenon is also seen in the Chinese Han subjects with T2D (e.g., *rs1801261*) (30). These findings suggest that the 3 *KATP* variants can be targeted to evaluate the effectiveness of hypoglycemic medications in the Chinese in addition to being latent genetic predisposition markers for T2D risk.

Currently, the mechanism by which *KATP* variants induce high diabetes risk remains unclear, possibly involving inadequate insulin secretion, resistance to insulin, or (and) impaired glucagon secretion. First, the *KATP* mutations are associated with impaired glucose-induced insulin secretion in the Turks (e.g., *rs1799854*, *rs1799859*, and *rs5219*) (7), the Finns (e.g., *rs5219*) (31), and the French-Canadians (e.g., *rs2285676* and *rs11024273*) (32). Secondly, *KATP* variants associate with decreased insulin sensitivity in the Poles (e.g., *rs80356622* and *rs80356624*) (33), the Finns and the Americans (e.g., *rs5219*) (31). Lastly, *KATP rs5219* correlates with impaired glucose-induced suppression of glucagon secretion (34). What is more important is that both animal and clinical studies indicate that *KATP* variants manifest their deleterious effects at early stages in the progression of T2D (e.g., during the progression from normal blood glucose levels to impaired glucose tolerance) (35), especially under the influence of environmental factors, such as diet (e.g., *rs5219* and alcohol consumption in the Koreans) (36) and lifestyle (e.g., *rs3758947* and physical inactivity in the Finns) (37). Indeed, *R1420H* (*chr11:17417205C* > *T*) correlates with increased risk of T2D (by 2-fold) with a 7-year earlier onset age (14). A recent study shows that the Chinese Han subjects with *KATP rs5219* or*rs1801261* are more susceptible to the development of prediabetes (38).

### ATP-Sensitive Potassium Channels Variants and Arteriosclerosis Cardiovascular Disease Risk

Arteriosclerosis cardiovascular disease, such as ACS and stroke are the major macro-vascular complications in patients with T2D (39), and are the leading cause of increased mortality among diabetics, even in people who optimally control the common risk factors for ASCVD. Among the patients with ACS, those with T2D are particularly at an increased risk for recurrent cardiovascular events and premature death, especially in China (40). Our results show that the Chinese participants with high diabetic risk genotype (A-allele) of *rs2285676* present 37% increased the risk of new-onset/recurrent ACS after a median follow-up of 45.8-months. The T-allele of *rs141294036* is not a diabetic risk predisposition allele, but the Chinese subjects with this mutation are at the highest risk for new-onset/recurrent ACS (increased by 59%). On the other hand, the new-onset of stroke risk increases by 1.58-fold and 1.30-fold among the Chinese subjects expressing the high diabetic risk genotype (*CC*) of *rs1799858* and *rs141294036*, respectively, after a median follow-up of 50.8-months. However, the *CC* genotype of *rs1799858* and *rs141294036* correlates only with moderate and high risk of IS. In addition to being related to the occurrence risk, a recent study further showed that the *KATP* variants (e.g., rs2237982, rs2283261, rs3819521, and rs8192695) are also linked to the progression of vascular events, and are involved in abnormal transcription in the target-tissue and promoter/enhancer markers (41). Compared to the heritabilityof*rs141294036* for new-onset stroke risk (0.44%, Supplementary Table 6), the heritability of the loci for new-onset/recurrent ACS risk (1.34%, Supplementary Table 5) are much higher. These results suggest that the genotypes, *rs2285676* (*AA* + *AG*), *rs1799858* (*CC*), and *rs141294036* (*CC*) are optimal genetic markers for macro-vascular events in diabetic patients, especially *rs141294036* for a new-onset/recurrent ACS risk.

### ATP-Sensitive Potassium Channels Variants and Heart Failure Risk

Type 2 diabetes is a very important independent risk factor for HF. Previous studies show that *KATP rs5219* prevalence is increased in the American patients with HF (21) while 3 *KATP* variants are associated with left ventricular remodeling in ischemic HF subjects in Ukraine (*e.g., rs5215*, *rs5219* and *rs757110*), suggesting that the *KATP* mutations may correlate with increased HF risk. This study shows a 1.78-fold increase in HF among the Chinese subjects with *TT* + *CT* genotype of *rs1799858* after a median follow-up of 48.1-months. Compared to the association between *rs1799858* and HF, *rs141293036* (*TT* + *CT*) correlates with weaker HF incidence risk (only increased by 45%). The heritability of HF is heterogeneous, and the genetic characteristics of different HF subtypes are still poorly understood. Further analysis of HF subtypes first indicates that *rs1799858* (*TT* + *CT*) only correlates with a high risk of HFpEF while *rs141294036* (*TT* + *CT*) correlates with a moderate risk of HFmrEF. The heritability of *rs1799858* for a new-onset of HF risk is up to 3.48% (Supplementary Table 8), especially 2.78% for HFpEF (Supplementary Table 9). As far as the current HFpEF and HFmrEF prevention and treatment strategy are concerned, the “phenotype”- based targeted therapy is a more feasible priority strategy (42). Hence, these findings indicate that the *TT* + *CT* genotype of *rs1799858* and *rs141294036* can serve as a potential phenotypic marker for HFpEF and HFmrEF phenotypes, especially, *rs1799858*.

### ATP-Sensitive Potassium Channels Variants and Atrial Fibrillation Risk

Type 2 diabetes is also one of the strongest independent risk factors for AF. However, the relationships between *KATP* variants and AF risk in the Chinese diabetic subjects remain unclear. This study shows that the Chinese subjects with high diabetic risk genotype (*CC*) of *rs141294036* are at 1.42-fold more risk of having AF at the time of enrollment. The T-allele of *rs1799858* does not correlate with diabetes risk, but the Chinese subjects with the mutation are at a higher risk of AF (approximately increased by 1.15-fold). After a median follow-up of 50.1-months, a similar association between the two variants and the risk of a new-onset of AF was found, where the AF risk was more than doubled in the presence of any of these variants (Supplementary Table 11). In this study, the total heritability of *rs1799858* and *rs141294036* for total AF risk reaches up to 1.85% (Supplementary Table 12). Similarly, an isolated study indicates that the *KATP* variant, *rs387906805* correlates with AF originating from the vein of Marshall (43). A large sample study also indicates an association of *KATP rs72554071* with increased AF susceptibility among the Americans based on 8-years follow-up (22). The pathogenic mechanism involves shortening of the action potential duration mediated by a transient outward current (22). These results suggest that *rs141294036* (*CC*) may be a preferred predisposition marker for diabetic AF in the Chinese subjects.

### ATP-Sensitive Potassium Channels Variants and Glucose and Lipid Metabolism Disorder/Chronic Inflammation

Glucose and lipid metabolism disorders, along with chronic low-grade inflammation, are essential in the initiation and progression of diabetic cardiovascular events. Our study and many other studies, demonstrates the association of *KATP* variants with glucose metabolism disorders; however, lipid metabolism disorders and inflammation are rarely highlighted. This study indicates that the three diabetic-risk related *KATP* variants are indeed correlated with dyslipidemia but exhibited heterogeneity as follows: none of the 3 *KATP* variants correlates with increased serum TC levels (Supplementary Table 19), and decreased serum HDL-L level (Supplementary Table 20). *KATP rs2285676* (*A*-allele) correlates with a higher risk of all subtypes [e.g., TRIG, LDL-C, ApoB and Lp(a)] of dyslipidemia except HDL-C and ApoA-I. The high diabetic risk genotype (*CC*) of *rs1799858* and *rs141294036*only correlates with moderate risk of increased serum Lp(a) levels while its counterpart genotype (*TT* + *CT*) correlates with a moderate risk of decreased serum ApoA-I levels. Besides ApoA-I, the *KATP rs1799858* (*TT* + *CT*) correlates with a high risk of increased serum LDL-C levels. On the other hand, the three diabetic-risk related *KATP* variants are associated with a moderate risk of low-grade inflammation levels (HsCRP ≥ 3.0 mg/L).

The clinical heterogeneity of diabetic cardiovascular phenotypes may relate to the combinations of hyperglycemia, different subtypes of dyslipidemia, and chronic low-grade inflammation (44). First, the *CC* genotype of *rs1799858* and *rs141294036* correlate with a high IS risk. These two variants correlate with a high risk of higher serum Lp(a) levels. In addition, two recent large sample studies confirm that high serum concentration of Lp(a) is associated with an increased risk of IS, both observationally and causally based on human genetics (45, 46). Second, the TT + CT genotype of *rs1799858* and *rs141294036* correlate with HFpEF and HFmrEF risks. Both the variants correlate with a high risk of decreased serum in the ApoA-I level. Among the lipoprotein components related to HDL-C, ApoA-I presents the greatest independent risk factor for cardiovascular risk events and is a better predictive marker for HF (47). In mouse models of HF, a reconstituted HDL-C improves the outcomes, especially HFpEF (48). Importantly, under the same adverse effect of HF incidence rate (e.g., 10%), HF occurs earlier (about 1 year; Supplementary Figure 4) in subjects with *TT* + *CT* genotype of *rs1799858* compared to those with *TT* + *CT* genotype of *rs141294036*. Besides the high risk of decreased serum ApoA-I level, *rs1799858* is also related to the coexisting elevated risk of high serum levels of LDL-C and HsCRP. Third, *rs2285676* (*AA* + *AG*) and *rs141294036* (*TT* + *CT*) are associated with ACS risk. The difference between them is that the former almost correlates with a higher risk of all subtypes of dyslipidemia except HDL-C and ApoA-I, where the latter shows the complete opposite. A consensus is established on the relationship between arteriosclerotic cholesterol [e.g., TRIG, LDL-C, ApoB and Lp(a)] and ACS risk, but little is known about its relationship with ApoA-I. Lower serum ApoA-I levels are associated with increased ACS risk and is manifested in individuals with a high cardiovascular risk (49). However, coronary atherosclerosis is not reduced by ApoA-I mimetic infusions in patients with ACS and high plaque burden (50). Lastly, *rs1799858* (*TT* + *CT*) and *rs141294036* (*CC*) are associated with AF risk. Both of them are associated with a high risk of low-grade inflammation (HsCRP ≥ 3.0 mg/L). A recent study shows that chronic low-grade inflammation correlates with increased AF risk in the Chinese participants having a metabolic syndrome (51).

### Strengths and Limitations

This study is the first long follow-up assessment of the genotype-cardiovascular phenotype profiles of *KATP* variants among diabetic subjects in China. Several limitations worth mentioning are as follows: First, the study determines the above associations successfully, but the direct evidence with regards to its pathophysiology is yet to be investigated. Second, two potential factors may influence the associations, including the sample size and cardiovascular events (e.g., stroke and HF) during the follow-up. Importantly, it is necessary to explore the association of glycemic parameters, such as HbA1C and FBG with the new onset of cardiovascular phenotypes, given there is a strong association between the glycemic control and macro-vascular complications. Initially, the current work only demonstrates the association between *KATP* and diabetic cardiovascular phenotypes. These results and plans should be further validated by large-scale, prospective cohort studies with large-scale sample size. Third, there is still a risk of potential Type I error so that Bonferroni adjustment is carried out to correct the significant thresholds. Fourth, microvascular dysfunction (MVD) in T2D is present in many tissues or organs and it manifests itself as a spectrum of specific phenotypes: LCI in the brain (52), HFpEF in the heart (53), and microalbuminuria in the kidney (54). We observed a potential clinical association between *KATP* genetic variants and MVD-related phenotypes (e.g., LCI and HFpEF), but the direct evidence based on MVD as the pathophysiological entity is still lacking and needs further investigation. Lastly, the crosstalk between the environmental and genetic factors results in the development of diabetes and its associated CVDs. Additionally, the effect of non-coding RNA on the pathophysiological processes from elevated serum glucose levels to different diabetic cardiovascular phenotypes needs further investigation.

## Conclusion

The *KATP* variants, *rs2285676*, *rs1799858*, and *rs141294036* are associated with increased risks of T2D and their related cardiovascular events (ACS, IS, HF, and AF) but show heterogeneity. These novel findings suggest that the three variants may serve as promising phenotypic markers of diabetic cardiovascular events. These markers are potentially valuable for early and “genotype-phenotype”-oriented prevention and treatment strategies.

## Data Availability Statement

The original contributions presented in the study are included in the article/Supplementary Material, further inquiries can be directed to the corresponding author/s

## Ethics Statement

The study received ethics approval from the Institutional Review Board of Guangzhou First People’s Hospital, South China University of Technology (K-2017-043-02). All procedures performed in studies involving human participants were in accordance with the ethics guidelines of the institutional and with the principles of the Declaration of Helsinki. Written consent has been obtained from each patient or subject after full explanation of the purpose and nature of all procedures used. The patients/participants provided their written informed consent to participate in this study.

## Author Contributions

CL: literature search, study format, writing protocol, collecting data, processing data, data interpretation, analyzing data, and writing manuscript. YL and JP: study format, writing protocol, recruiting patients, data interpretation, and carrying out the molecular genetics. TG, JZ, SY, and YS: recruiting patients, following up patients, collecting data. DW: echocardiography testing. All authors read and approved the final manuscript.

## Conflict of Interest

The authors declare that the research was conducted in the absence of any commercial or financial relationships that could be construed as a potential conflict of interest.

## Publisher’s Note

All claims expressed in this article are solely those of the authors and do not necessarily represent those of their affiliated organizations, or those of the publisher, the editors and the reviewers. Any product that may be evaluated in this article, or claim that may be made by its manufacturer, is not guaranteed or endorsed by the publisher.

## Footnotes

1
https://ldlink.nci.nih.gov/?tab=ldmatrix

## Abbreviations

ACE: angiotensin converting enzyme
ACS: acute coronary syndrome
AF: atrial fibrillation
Alb: albumin
ALD: aldosterone
ALT: alanine aminotransferase
Ang I/II: angiotensin I/II
ApoA-I: apolipoproteinA-I
ApoB: apolipoproteinB
ASCVD: arteriosclerosis cardiovascular disease
AST: aspartate aminotransferase
BMI: body mass index
BUN: blood urea nitrogen
CAD: coronary atherosclerotic heart disease
Ce-MVD: cerebral microvascular dysfunction
CI: confidence interval
Co-MVD: coronary microvascular dysfunction
CS: cardiogenic stroke
DBP: diastolic blood pressure
HTN: Hypertension
FBG: fasting blood glucose
HbA1C: glycosylated haemoglobin
HDL-C: high-density lipoprotein cholesterol
HF: Heart failure
HFpEF: HF with preserved ejection fraction
HFmrEF: HF with mildly reduced ejection fraction
HFrEF: HF with reduced ejection fraction
HGB: hemoglobin concentration
HS: hemorrhagic stroke
HR: hazard ratio
HsCRP: high-sensitivity C-reactive protein
IS: ischemic stroke
K^+^: serum potassium
KATP: ATP-sensitive potassium channels
Kir6.x: inward-rectifier potassium channel
LAD: left atrial end-diastolic dimension
LCI: lacunar cerebral infarct
LD: linkage disequilibrium
LDL-C: low-density lipoprotein cholesterol
Lp(a): lipoprotein(a)
LVD: left ventricular end-diastolic diameter
LVEF: left ventricular ejection fraction
LVMI: left ventricular mass index
MAF: minor allele frequency
MVD: microvascular dysfunction
Na^+^: serum sodium
OR: odds ratio
P2hBS: postprandial blood glucose 2 h
PLT: platelet count
RAAS: renin-angiotensin-aldosterone system
RAD: right atrial end-diastolic dimension
RVD: right ventricular end-diastolic diameter
SBP: systolic blood pressure
Scr: serum creatinine
SNP: single nucleotide polymorphism
SUR: sulfonylurea receptor
TC: total cholesterol
T2D: type 2 diabetes
TRIG: triglyceride
UA: serum uric acid
WBC: white blood cell count.
