# Supplementary material for: Associations of ATP-Sensitive Potassium Channel’s Gene Polymorphisms With Type 2 Diabetes and Related Cardiovascular Phenotypes
Source: Front Cardiovasc Med. 2022 Mar 23;9:816847. doi: 10.3389/fcvm.2022.816847 (PMC8984103; doi:10.3389/fcvm.2022.816847)
Supplement: Supplementary file 1 [file Data_Sheet_1.docx]

**Supplementary material**

**Additional Results**

***KATP* SNPs and genotype frequencies**

As shown in Table S2, *KATP* SNPs rs2285676 (*P*=0.249), rs11046182 (*P*=0.061), rs1799858 (*P*=0.075) and rs141294036 (*P*=0.115) examined followed to the Hardy-Weinberg equilibrium (HWE). The power of the 4 KATP variants was no less than 0.8 in this study.

**Association of *KATP* SNPs with LCI risk**

As shown in Table S13, only *KATP* rs1799858 (CC, adjusted OR=2.27, 95% CI: 1.10-4.71, *P*=0.027) were associated with high risk of LCI at enrollment.

**Association of *KATP* SNPs with increased serum TC level (≥ 4.0 mmol/L) in study participants.**

As shown in Table S19, these 9 *KATP* SNPs was not associated with increased higher serum TC level (≥ 4.0 mmol/L, all adjusted *P*>0.05).

**Association of *KATP* SNPs with decreased serum HDL-C level (< 1.0 mmol/L) in study participants.**

As shown in Table S20, these 9 *KATP* SNPs was not associated with decreased serum HDL-C level (< 1.0 mmol/L, all adjusted *P*>0.05).

**Table legends**

Table S1. The primers of *KATP* SNPs in the Sequenom MassARRAY system.

Table S2. Descriptive information on *KATP* SNPs and genotype frequencies (Hardy-Weinberg equilibrium) in study participants.

Table S3. Partial clinical characteristics of study participants at the end of the follow-up.

Table S4. Association of *KATP* SNPs with T2D in study participants.

Table S5. Association of *KATP* SNPs with new-onset/recurrent ACS risk in study participants.

Table S6. Association of *KATP* SNPs with new-onset stroke risk in study participants.

Table S7. Association of *KATP* SNPs with different types of stroke in study participants.

Table S8. Association of *KATP* SNPs with new-onset HF risk in study participants.

Table S9. Association of *KATP* SNPs with different types of HF in study participants.

Table S10. Association of *KATP* SNPs with AF risk at enrollment in study participants.

Table S11. Association of *KATP* SNPs with new-onset AF risk in study participants.

Table S12. Association of *KATP* SNPs with total AF risk in study participants.

Table S13. Association of *KATP* SNPs with LCI risk at enrollment in study participants.

Table S14. Association of *KATP* SNPs with increased serum TRIG level (≥ 1.70 mmol/L) in study participants.

Table S15. Association of *KATP* SNPs with increased serum LDL-C level (≥ 1.40 mmol/L) in study participants.

Table S16. Association of *KATP* SNPs with increased serum ApoB level (≥ 80 mg/dL) in study participants.

Table S17. Association of *KATP* SNPs with decreased serum ApoA-I level (< 120 mg/dL) in study participants.

Table S18. Association of *KATP* SNPs with increased serum Lp(a) level (≥ 300 mg/dL) in study participants.

Table S19. Association of *KATP* SNPs with increased serum TC level (≥ 4.0 mmol/L) in study participants.

Table S20 Association of *KATP* SNPs with decreased serum HDL-C level (< 1.0 mmol/L) in study participants.

Table S21. Association of *KATP* SNPs with increased serum HsCRP level (≥ 3.0 mg/L) in study participants.

**Figure legends**

Figure S1. Schematic representation on the structure, location of polymorphic sites and LD plot of 9 *KATP* SNPs in Chinese (CHS + CHB).

Figure S2. Association of *KATP* SNPs with new-onset/recurrent ACS in study participants.

Figure S3. Association of *KATP* SNPs with new-onset/recurrent stroke in study participants.

Figure S4. Association of *KATP* SNPs with incident HF in study participants.

Figure S5. Association of *KATP* SNPs with new-onset AF in study participants.

**Table S1. The primers of *KATP* SNPs in the Sequenom MassARRAY system.**

| **NQ** | **SNP_ID** | **Gene**  **(Protein)** | **Promoter**  **histone marks** | **Enhancer**  **histone marks** | **DNAse** | **Motifs**  **changed** | **Selected eQTL hits** | **dbSNP function** | **Primer^b^** | |
| --- | --- | --- | --- | --- | --- | --- | --- | --- | --- | --- |
| 1 | *rs2285676* | KCNJ11  (Kir 6.2) | 5 tissues | 10 tissues | 8 tissues | E2A,Lmo2-complex,TCF12 | 14 hits | 3'-UTR | Primer 1: | ACGTTGGATGACACCCTCTCTCATCAACTG |
|  |  |  |  |  |  |  |  |  | Primer 2: | ACGTTGGATGGCTCTACTTGGTCCCTGAAA |
|  |  |  |  |  |  |  |  |  | Primer 3: | CCTCCCTGAAAAAGCACC |
| 2 | *rs11046182* | KCNJ8  (Kir 6.1) | - | HRT, SKIN | - | Pax-5,RXRA | 2 hits | intronic | Primer 1: | ACGTTGGATGAGATTCTTACAAGGAGCCCG |
|  |  |  |  |  |  |  |  |  | Primer 2: | ACGTTGGATGTCTCATAGGAGTGTGAACCC |
|  |  |  |  |  |  |  |  |  | Primer 3: | CCCTACGGTGAACTG |
| 3 | *rs1799858* | ABCC8  (SUR1) | - | MUS, SPLN, BLD | THYM | - | 1 hits | missense | Primer 1: | ACGTTGGATGTGAGGCCCCGACAATCCTCC |
|  |  |  |  |  |  |  |  |  | Primer 2: | ACGTTGGATGAGTGGGTCCTCACCTCCAAA |
|  |  |  |  |  |  |  |  |  | Primer 3: | GCCACTCAGGGTTGTGAACCGCAA |
| 4 | *rs4148671* | ABCC9  (SUR2) | - | - | - | Cdc5,Pou3f2,SETDB1 | - | intronic | Primer 1: | ACGTTGGATGCTTCAAGGATTTATTTCCCC |
|  |  |  |  |  |  |  |  |  | Primer 2: | ACGTTGGATGAGGTGTAAGTCAAGTAACTC |
|  |  |  |  |  |  |  |  |  | Primer 3: | GGGGCAAGTAACTCAAGGAAAGATG |
| 5 | *rs78148713* | KCNJ8/ABCC9 (Kir 6.1/SUR2) | - | 4 tissues | BLD,  HRT | Sox | - | intronic | Primer 1: | ACGTTGGATGAAGTGGAAGCTGCATGAGAG |
|  |  |  |  |  |  |  |  |  | Primer 2: | ACGTTGGATGTACTCTTGGGATCTCGGAAC |
|  |  |  |  |  |  |  |  |  | Primer 3: | CCACTCTTGGGATCTCGGAACAATTTG |
| 6 | *rs145456027* | KCNJ8/ABCC9 (Kir 6.1/SUR2) | - | - | - | - | - | intronic | Primer 1: | ACGTTGGATGCAAAGCTGTAGGCATCACAC |
|  |  |  |  |  |  |  |  |  | Primer 2: | ACGTTGGATGGTACCAGTACCTTGCTGTTC |
|  |  |  |  |  |  |  |  |  | Primer 3: | GGCTTTTCTGGTTACTGTAGCCTTGTAG |
| 7 | *rs147265929* | KCNJ11/ABCC8 (Kir 6.2/SUR1) | - | ESDR, BLD | ESDR | - | - | intronic | Primer 1: | ACGTTGGATGTTCCTTTCCGAGCTTCTCTG |
|  |  |  |  |  |  |  |  |  | Primer 2: | ACGTTGGATGAGAAAAGCCCACCAGTTATC |
|  |  |  |  |  |  |  |  |  | Primer 3: | GAGCGGCCCACCAGTTATCGGAGGC |
| 8 | *rs61928479* | ABCC9  (SUR2) | - | FAT, GI | - | - | - | intronic | Primer 1: | ACGTTGGATGGAAGTCTATTTACTGGGCCG |
|  |  |  |  |  |  |  |  |  | Primer 2: | ACGTTGGATGATCTCCTGACCTTGTGATCC |
|  |  |  |  |  |  |  |  |  | Primer 3: | GGGATTACAGGCGTG |
| 9 | *rs141294036* | KCNJ8/ABCC9 (Kir 6.1/SUR2) | - | - | LNG | 4 altered motifs | - | intronic | Primer 1: | ACGTTGGATGAAACCACCTACTCAAGCCTC |
|  |  |  |  |  |  |  |  |  | Primer 2: | ACGTTGGATGACAGCAGTCTGAAGTTGAGC |
|  |  |  |  |  |  |  |  |  | Primer 3: | CCTCGTGGTGGGAGGAGGGGC |

Types of primer: Primer 1,1st- forward PCR primer (5’-3’); Primer 2, 2nd- reverse PCR primer (5’-3’), and Primer 3, extension primer (5’-3’)

**Table S2. Descriptive information on *KATP* SNPs and genotype frequencies (Hardy-Weinberg equilibrium) in study participants.**

| **NQ** | **KATP SNPs** | | **MAF in CHB^a^** | **Minor Allele** | **Dominant Model** | **Non-T2D group (N=634)** | | |  | **T2D group (N=636)** | | | **Power** |
| --- | --- | --- | --- | --- | --- | --- | --- | --- | --- | --- | --- | --- | --- |
|  |  |  |  |  |  | **Frequencies (N/%)** | **MAF^b^** | ***P_HWE_* value^c^** |  | **Frequencies (N/%)** | **MAF^b^** | ***P_HWE_* value^d^** |  |
| 1 | *rs2285676* | *GG* | 0.451 | *A* | *AA+GA vs. GG* | 248(39.1) | 0.383 | 0.249 |  | 204(32.1) | 0.425 | 0.282 | 0.999 |
|  |  | *GA* |  |  |  | 286(45.1) |  |  |  | 324(50.9) |  |  |  |
|  |  | *AA* |  |  |  | 100(15.8) |  |  |  | 108(17.0) |  |  |  |
| 2 | *rs11046182* | *GG* | 0.180 | *A* | *AA+GA vs. GG* | 390(61.5) | 0.224 | 0.061 |  | 418(65.7) | 0.193 | 0.284 | 0.999 |
|  |  | *GA* |  |  |  | 204(32.2) |  |  |  | 190(29.9) |  |  |  |
|  |  | *AA* |  |  |  | 40(6.3) |  |  |  | 28(4.4) |  |  |  |
| 3 | *rs1799858* | *CC* | 0.165 | *T* | *TT+CT vs. CC* | 374(59.0) | 0.224 | 0.075 |  | 426(67.0) | 0.187 | 0.135 | 0.999 |
|  |  | *CT* |  |  |  | 236(37.2) |  |  |  | 182(28.6) |  |  |  |
|  |  | *TT* |  |  |  | 52(3.8) |  |  |  | 28(4.4) |  |  |  |
| 4 | *rs4148671* | *CC* | 0.063 | *T* | *TT+CT vs. CC* | 562(88.6) | 0.066 | <0.001 |  | 544(88.6) | 0.083 | 0.006 | 0.120 |
|  |  | *CT* |  |  |  | 60(9.5) |  |  |  | 78(12.3) |  |  |  |
|  |  | *TT* |  |  |  | 12(1.9) |  |  |  | 14(2.2) |  |  |  |
| 5 | *rs78148713* | *CC* | 0.053 | *C* | *CC+CT vs. TT* | 14(2.2) | 0.035 | <0.001 |  | 4(0.6) | 0.025 | 0.001 | 0.121 |
|  |  | *CT* |  |  |  | 16(2.5) |  |  |  | 24(3.8) |  |  |  |
|  |  | *TT* |  |  |  | 604(95.3) |  |  |  | 608(95.6) |  |  |  |
| 6 | *rs145456027* | *CC* | 0.068 | *C* | *CC+CT vs. TT* | 10(1.6) | 0.028 | <0.001 |  | 8(1.3) | 0.014 | 0.002 | 0.209 |
|  |  | *CT* |  |  |  | 16(2.5) |  |  |  | 2(0.3) |  |  |  |
|  |  | *TT* |  |  |  | 608(95.9) |  |  |  | 626(98.4) |  |  |  |
| 7 | *rs147265929* | *GG* | 0.053 | *G* | *GG+GT vs. TT* | 6(0.9) | 0.046 | 0.014 |  | 4(0.6) | 0.041 | 0.003 | 0.055 |
|  |  | *GT* |  |  |  | 46(7.3) |  |  |  | 44(6.9) |  |  |  |
|  |  | *TT* |  |  |  | 582(91.8) |  |  |  | 588(92.5) |  |  |  |
| 8 | *rs61928479* | *AA* | 0.068 | *T* | *TT+AT vs. AA* | 52(8.2) | 0.481 | <0.001 |  | 86(13.5) | 0.451 | <0.001 | 0.999 |
|  |  | *AT* |  |  |  | 554(87.4) |  |  |  | 526(82.7) |  |  |  |
|  |  | *TT* |  |  |  | 28(4.4) |  |  |  | 24(3.8) |  |  |  |
| 9 | *rs141294036* | *CC* | 0.073 | *T* | *TT+CT vs. CC* | 214(32.8) | 0.406 | 0.115 |  | 270(42.5) | 0.366 | 0.012 | 0.999 |
|  |  | *CT* |  |  |  | 325(51.3) |  |  |  | 266(41.8) |  |  |  |
|  |  | *TT* |  |  |  | 95(14.9) |  |  |  | 100(15.7) |  |  |  |

^a^CHB：Han Chinese in Beijing, China；^b^MAF: minor allele frequency; ^c^*P*_HWE_ value for subjects without T2D (control); ^d^*P*_HWE_ value for subjects with T2D.

**Table S3. Partial clinical characteristics of study participants at the end of the follow-up.**

|  | | Non-T2D | T2D | ***P* value** |
| --- | --- | --- | --- | --- |
| Sample (N) | | 634 | 636 | - |
| **Medical condition** | |  |  |  |
| HTN (N/%) | | 248(39.1) | 279(43.9) | 0.086 |
| NYHA (N/%) | |  |  |  |
|  | I | 310(48.9) | 276(43.4) | 0.148 |
|  | II | 280(44.2) | 308(48.4) |  |
|  | III | 38(6.0) | 40(6.3) |  |
|  | IV | 6(0.9) | 12(1.9) |  |
| New-onset/recurrent ACS (N/%) | | 194(30.6) | 222(24.9) | 0.102 |
| New-onset stroke (N/%) | | 26(4.1) | 32(5.0) | 0.427 |
| Stroke subtypes (N/%) | |  |  |  |
|  | Non-stroke | 608(95.9) | 604(95.0) | 0.275 |
|  | HS | 1(0.2) | 4(0.6) |  |
|  | IS | 20(3.2) | 26(4.1) |  |
|  | CS | 5(0.8) | 2(0.3) |  |
| **HF (N/%)** | | **120(18.9)** | **162(25.5)** | **0.005** |
| **HF subtypes (N/%)** | |  |  |  |
|  | **Non-HF** | **514(81.1)** | **474(74.5)** | **0.013** |
|  | **HFpEF** | **82(12.9)** | **104(16.4)** |  |
|  | **HFmrEF** | **16(2.5)** | **34(5.3)** |  |
|  | **HFrEF** | **22(3.5)** | **24(3.8)** |  |
| New-onset AF (N/%) | | 44(6.9) | 50(7.9) | 0.530 |
| **Combined medication** | |  |  |  |
| (A)Antiplatelet drugs (N/%) | | 564(89.0) | 582(91.2) | 0.126 |
| (B)Warfarin (N/%) | | 24(3.8) | 24(3.8) | 0.991 |
| (C) Statins (N/%) | | 566(89.3) | 586(92.1) | 0.079 |
| **(D) RSIs (N/%)** | | **290(45.7)** | **384(60.4)** | **<0.001** |
| **(E) BBs (N/%)** | | **406(64.0)** | **448(70.4)** | **0.015** |
| **(F) MRAs (N/%)** | | **128(20.2)** | **158(24.8)** | **0.047** |
| (G) CCBs (N/%) | | 122(19.2) | 148(23.3) | 0.079 |
| (H) Diuretics (N/%) | | 138(21.8) | 168(26.4) | 0.053 |
| (J) Digoxin (N/%) | | 64(10.1) | 66(10.4) | 0.868 |
| (K) Nitrates (N/%) | | 66(10.4) | 76(11.9) | 0.384 |

**Table S4. Association of *KATP* SNPs with T2D in study participants.**

| ***KATP* SNPs** | | **T2D (N/%)** | | ***χ2*** | ***P* value** | **Crude**  **OR (95% CI)** | **Crude**  ***P* value** | **Adjusted**  **OR (95% CI)**^a^ | **Adjusted**  ***P* value**^a^ | **Adjusted**  **OR (95% CI)^b^** | **Adjusted**  ***P* value^b^** | **Explained variance (%)^b^** |
| --- | --- | --- | --- | --- | --- | --- | --- | --- | --- | --- | --- | --- |
|  |  | **NO** | **YES** |  |  |  |  |  |  |  |  |  |
| *rs2285676* | *GG* | 248(39.1) | 204(32.1) | 6.867 | 0.009 | 1.00 |  | 1.00 |  | 1.00 |  |  |
|  | *AA+GA* | 386(60.9) | 432(67.9) |  |  | **1.36(1.08-1.71)** | **0.009** | **1.41(1.12-1.79)** | **0.004** | **1.43(1.13-1.81)** | **0.003** | **0.68** |
| *rs11046182* | *GG* | 390(61.5) | 418(65.7) | 2.430 | 0.119 | 1.19(0.94-1.50) | 0.146 | 1.21(0.95-1.53) | 0.118 | 1.23(0.97-1.56) | 0.083 | - |
|  | *AA+GA* | 244(38.5) | 218(34.3) |  |  | 1.00 |  | 1.00 |  | 1.00 |  |  |
| *rs1799858* | *CC* | 374(59.0) | 426(67.0) | 8.696 | 0.003 | **1.41(1.12-1.77)** | **0.003** | **1.42(1.12-1.78)** | **0.003** | **1.42(1.12-1.78)** | **0.004** | **0.65** |
|  | *TT+CT* | 260(41.0) | 210(33.0) |  |  | 1.00 |  | 1.00 |  | 1.00 |  |  |
| *rs4148671* | *CC* | 562(88.6) | 544(85.5) | 2.729 | 0.099 | 1.00 |  | 1.00 |  | 1.00 |  |  |
|  | *TT+CT* | 72(11.4) | 92(14.5) |  |  | 1.32(0.95-1.84) | 0.099 | 1.26(0.90-1.76) | 0.171 | 1.22(0.87-1.71) | 0.241 | - |
| *rs78148713* | *CC+CT* | 30(4.7) | 28(4.4) | 0.079 | 0.779 | 1.00 |  | 1.00 |  | 1.00 |  |  |
|  | *TT* | 604(95.3) | 608(95.6) |  |  | 1.08(0.64-1.83) | 0.779 | 1.11(0.66-1.90) | 0.689 | 1.17(0.69-2.01) | 0.558 | - |
| *rs145456027* | *CC+CT* | 26(4.1) | 10(1.6) | 7.371 | 0.007 | 1.00 |  | 1.00 |  | 1.00 |  |  |
|  | *TT* | 608(95.9) | 626(98.4) |  |  | 2.68(1.28-5.60) | 0.009 | 2.75(1.31-5.78) | 0.008 | 2.94(1.39-6.22) | 0.005 | - |
| *rs147265929* | *GG+GT* | 52(8.2) | 48(7.5) | 0.188 | 0.665 | 1.00 |  | 1.00 |  | 1.00 |  |  |
|  | *TT* | 582(91.8) | 588(92.5) |  |  | 1.10(0.73-1.65) | 0.665 | 1.11(0.73-1.67) | 0.632 | 1.12(0.74-1.71) | 0.591 | - |
| *rs61928479* | *AA* | 52(8.2) | 86(13.5) | 9.278 | 0.002 | 1.75(1.22-2.52) | 0.003 | 1.72(1.19-2.48) | 0.004 | 1.71(1.18-2.47) | 0.005 | - |
|  | *TT+AT* | 582(91.8) | 550(86.5) |  |  | 1.00 |  | 1.00 |  | 1.00 |  |  |
| *rs141294036* | *CC* | 214(33.8) | 270(42.5) | 10.186 | 0.001 | **1.45(1.15-1.82)** | **0.001** | **1.44(1.15-1.81)** | **0.002** | **1.45(1.15-1.83)** | **0.002** | **0.77** |
|  | *TT+CT* | 420(66.2) | 366(57.5) |  |  | 1.00 |  | 1.00 |  | 1.00 |  |  |

^a^**Model 1**: After adjustment for gender, age, smoking, alcohol consumption and BMI.

^b^**Model 1s**: It is the same as Model 1, and also including SBP, DBP, HsCRP and RAAS activity (ACE, renin, Ang I, Ang II and ALD).

**Table S5. Association of *KATP* SNPs with new-onset/recurrent ACS risk in study participants.**

| ***KATP* SNPs** | | **New-onset/recurrent ACS (N/%)** | | ***χ2*** | ***P* value** | **Crude**  **OR (95% CI)** | **Crude**  ***P* value** | **Adjusted**  **OR (95% CI)^a^** | **Adjusted**  ***P* value^a^** | **Adjusted**  **OR (95% CI)^b^** | **Adjusted**  ***P* value^b^** | **Explained variance (%)^b^** |
| --- | --- | --- | --- | --- | --- | --- | --- | --- | --- | --- | --- | --- |
|  |  | **NO** | **YES** |  |  |  |  |  |  |  |  |  |
| *rs2285676* | *GG* | 318(37.2) | 134(32.2) | 3.081 | 0.079 | 1.00 |  | 1.00 |  | 1.00 |  |  |
|  | *AA+GA* | 536(62.8) | 282(67.8) |  |  | 1.19(0.97-1.47) | 0.093 | **1.32(1.06-1.63)** | **0.012** | **1.37(1.10-1.70)** | **0.005** | **0.61** |
| *rs11046182* | *GG* | 542(63.5) | 266(63.9) | 0.027 | 0.868 | 1.00(0.82-1.22) | 0.988 | 1.01(0.82-1.24) | 0.911 | 1.03(0.84-1.27) | 0.783 | - |
|  | *AA+GA* | 312(36.5) | 150(36.1) |  |  | 1.00 |  | 1.00 |  | 1.00 |  |  |
| *rs1799858* | *CC* | 544(63.7) | 256(61.5) | 0.561 | 0.454 | 0.94(0.78-1.15) | 0.568 | 0.95(0.77-1.17) | 0.628 | 0.94(0.76-1.17) | 0.576 | - |
|  | *TT+CT* | 310(36.3) | 160(38.5) |  |  | 1.00 |  | 1.00 |  | 1.00 |  |  |
| *rs4148671* | *CC* | 752(88.1) | 354(85.1) | 2.179 | 0.140 | 1.00 |  | 1.00 |  | 1.00 |  |  |
|  | *TT+CT* | 102(11.9) | 62(14.9) |  |  | 1.23(0.94-1.61) | 0.131 | 1.18(0.85-1.62) | 0.320 | 1.17(0.84-1.62) | 0.349 | - |
| *rs78148713* | *CC+CT* | 34(4.0) | 28(4.4) | 2.052 | 0.152 | 1.00 |  | 1.00 |  | 1.00 |  |  |
|  | *TT* | 820(96.0) | 392(94.2) |  |  | 0.73(0.48-1.10) | 0.134 | 0.70(0.43-1.13) | 0.148 | 0.73(0.45-1.18) | 0.199 | - |
| *rs145456027* | *CC+CT* | 32(3.7) | 4(1.0) | 7.881 | 0.005 | 1.00 |  | 1.00 |  | 1.00 |  |  |
|  | *TT* | 822(96.3) | 412(99.0) |  |  | 3.40(1.27-9.11) | 0.015 | 2.45(0.90-6.71) | 0.081 | 2.36(0.86-6.44) | 0.094 | - |
| *rs147265929* | *GG+GT* | 72(8.4) | 28(6.7) | 1.115 | 0.291 | 1.00 |  | 1.00 |  | 1.00 |  |  |
|  | *TT* | 782(91.6) | 388(93.3) |  |  | 1.18(0.80-1.73) | 0.397 | 0.82(0.55-1.24) | 0.347 | 0.77(0.51-1.16) | 0.204 | - |
| *rs61928479* | *AA* | 96(11.2) | 42(10.1) | 0.379 | 0.538 | 0.90(0.65-1.24) | 0.513 | 0.80(0.57-1.11) | 0.180 | 0.76(0.54-1.06) | 0.110 | - |
|  | *TT+AT* | 758(88.8) | 374(89.9) |  |  | 1.00 |  | 1.00 |  | 1.00 |  |  |
| *rs141294036* | *CC* | 344(40.3) | 140(33.7) | 5.029 | 0.022 | 1.00 |  | 1.00 |  | 1.00 |  |  |
|  | *TT+CT* | 510(59.7) | 276(66.3) |  |  | **1.24(1.01-1.52)** | **0.036** | **1.54(1.24-1.92)** | **<0.001** | **1.59(1.28-1.99)** | **<0.001** | **1.34** |

^a^**Model 2**: After adjustment for gender, age, smoking, alcohol consumption, BMI, WBC, blood glucose levels (FBS, P2hBS and HbA1C), liver function (ALT, AST and Alb), renal function (Scr, BUN and UA), serum sodium and potassium levels, HsCRP, HbA1C, RAAS activity (ACE, renin, Ang I, Ang II and ALD), dyslipidemia [TRIG, TC, LDL-C, ApoB, HDL-C, ApoA-I and Lp(a)], medical condition (T2D, EH, HF and AF), NYHA functional classification, and echocardiography index (RVD, RAD, LVD, LAD, LVMI, and LVEF).

^b^**Model 2s**: It is the same as Model 2, and also including combined medication, such as antiplatelet drugs, warfarin, statins, RSIs, BBs, MRA, CCBs, diuretics, digoxin, nitrates, and hypoglycemic agents.

**Table S6. Association of *KATP* SNPs with new-onset stroke risk in study participants.**

| ***KATP* SNPs** | | **New-onset** **stroke (N/%)** | | ***χ2*** | ***P* value** | **Crude**  **OR (95% CI)** | **Crude**  ***P* value** | **Adjusted**  **OR (95% CI)^a^** | **Adjusted**  ***P* value^a^** | **Adjusted**  **OR (95% CI)^b^** | **Adjusted**  ***P* value^b^** | **Explained variance (%)^b^** |
| --- | --- | --- | --- | --- | --- | --- | --- | --- | --- | --- | --- | --- |
|  |  | **NO** | **YES** |  |  |  |  |  |  |  |  |  |
| *rs2285676* | *GG* | 434(35.8) | 18(31.0) | 0.550 | 0.458 | 1.00 |  | 1.00 |  | 1.00 |  |  |
|  | *AA+GA* | 778(64.2) | 40(69.0) |  |  | 1.20(0.69-2.10) | 0.519 | 0.98(0.52-1.87) | 0.961 | 1.13(0.58-2.24) | 0.716 | - |
| *rs11046182* | *GG* | 766(63.2) | 42(72.4) | 2.030 | 0.154 | 1.53(0.86-2.72) | 0.150 | 1.83(0.95-3.50) | 0.069 | 1.68(0.86-3.26) | 0.127 | - |
|  | *AA+GA* | 446(36.8) | 16(27.6) |  |  | 1.00 |  | 1.00 |  | 1.00 |  |  |
| *rs1799858* | *CC* | 758(62.5) | 42(72.4) | 2.314 | 0.128 | 1.57(0.88-2.79) | 0.126 | **2.27(1.15-4.49)** | **0.019** | **2.58(1.22-5.43)** | **0.013** | **0.48** |
|  | *TT+CT* | 454(37.5) | 16(27.6) |  |  | 1.00 |  | 1.00 |  | 1.00 |  |  |
| *rs4148671* | *CC* | 1054(87.0) | 52(89.7) | 0.357 | 0.550 | 1.00 |  | 1.00 |  | 1.00 |  |  |
|  | *TT+CT* | 158(13.0) | 6(10.3) |  |  | 0.77(0.33-1.78) | 0.536 | 0.76(0.29-1.98) | 0.571 | 0.80(0.30-2.17) | 0.666 | - |
| *rs78148713* | *CC+CT* | 56(4.6) | 2(3.4) | 0.175 | 0.676 | 1.00 |  | 1.00 |  | 1.00 |  |  |
|  | *TT* | 1156(95.4) | 56(96.6) |  |  | 1.38(0.34-5.64) | 0.658 | 1.39(0.31-6.37) | 0.669 | 1.45(0.31-6.71) | 0.635 | - |
| *rs145456027* | *CC+CT* | 36(3.0) | 0(0.0) | 1.773 | 0.183 | - |  | - |  | - |  |  |
|  | *TT* | 1176(97.0) | 58(100.0) |  |  | - | - | - | - | - | - | - |
| *rs147265929* | *GG+GT* | 98(8.1) | 2(3.4) | 1.641 | 0.200 | 1.00 |  | 1.00 |  | 1.00 |  |  |
|  | *TT* | 1114(91.9) | 56(96.6) |  |  | 2.37(0.58-9.71) | 0.231 | 1.47(0.33-6.51) | 0.612 | 2.00(0.42-9.38) | 0.382 | - |
| *rs61928479* | *AA* | 132(10.9) | 6(10.3) | 0.017 | 0.896 | 0.94(0.41-2.20) | 0.892 | 0.80(0.29-2.24) | 0.672 | 1.02(0.35-3.03) | 0.965 | - |
|  | *TT+AT* | 1080(89.1) | 52(89.7) |  |  | 1.00 |  | 1.00 |  | 1.00 |  |  |
| *rs141294036* | *CC* | 458(37.8) | 26(44.8) | 1.163 | 0.281 | 1.34(0.80-2.24) | 0.272 | **2.07(1.08-3.96)** | **0.028** | **2.30(1.16-4.55)** | **0.017** | **0.44** |
|  | *TT+CT* | 754(62.2) | 32(55.2) |  |  | 1.00 |  | 1.00 |  | 1.00 |  |  |

^a^**Model 3**: After adjustment for gender, age, smoking, alcohol consumption, BMI, WBC, blood glucose levels (FBS, P2hBS and HbA1C), liver function (ALT, AST and Alb), renal function (Scr, BUN and UA), serum sodium and potassium levels, HsCRP, RAAS activity (ACE, renin, Ang I, Ang II and ALD), dyslipidemia [TRIG, TC, LDL-C, ApoB, HDL-C, ApoA-I and Lp(a)], medical condition (T2D, EH, CAD(ACS), AF, and LCI), NYHA functional classification, and echocardiography index (RVD, RAD, LVD, LAD, and LVEF).

^b^**Model 3s**: It is the same as Model 3, and also including combined medication, such as antiplatelet drugs, warfarin, statins, RSIs, BBs, MRA, CCBs, diuretics, digoxin, nitrates, and hypoglycemic agents.

**Table S7. Association of *KATP* SNPs with different types of stroke in study participants.**

| ***KATP* SNPs** | **Subtypes of stroke**  **(N/%)** | **Genotypes (N/%)** | | **Crude**  **OR (95% CI)** | ***P* value** | **Adjusted**  **OR (95% CI)^a^** | ***P* value^a^** | **Adjusted**  **OR (95% CI)^b^** | ***P* value^b^** | **Explained variance (%)^b^** |
| --- | --- | --- | --- | --- | --- | --- | --- | --- | --- | --- |
|  |  | **CC** | **TT+CT (Ref.)** |  |  |  |  |  |  |  |
| *rs1799858* | Non-stroke | 758(94.8) | 454(96.6) | Ref. | - | Ref. | - | Ref. | - |  |
|  | HS | 1(0.1) | 4(0.9) | 0.15(0.02-1.34) | 0.090 | 0.09(0.01-1.81) | 0.116 | 0.08(0.01-1.14) | 0.082 | - |
|  | IS | 37(4.6) | 9(1.9) | **2.46(1.18-5.15)** | **0.017** | **2.56(1.19-5.49)** | **0.016** | **2.38(1.11-5.10)** | **0.025** | **0.39** |
|  | CS | 4(0.5) | 3(0.6) | 0.80(0.18-3.58) | 0.769 | 0.82(0.13-5.10) | 0.827 | 0.29(0.03-2.68) | 0.273 | - |
| *rs141294036* | Non-stroke | 458(94.6) | 745(95.9) | Ref. | - | Ref. | - | Ref. | - | - |
|  | HS | 2(0.4) | 3(0.4) | 1.10(0.18-6.59) | 0.919 | 0.55(0.04-6.94) | 0.644 | 0.34(0.02-5.85) | 0.454 | - |
|  | IS | 23(4.8) | 23(2.9) | 1.65(0.91-2.97) | 0.097 | **2.06(1.09-3.90)** | **0.026** | **1.95(1.04-3.66)** | **0.037** | **0.34** |
|  | CS | 1(0.2) | 6(0.8) | 0.27(0.03-2.29) | 0.232 | 0.48(0.04-5.97) | 0.570 | 0.37(0.03-4.32) | 0.427 | - |

^a^**Model 4**: After adjustment for gender, age, smoking, alcohol consumption, BMI, WBC, blood glucose levels (FBS, P2hBS and HbA1C), liver function (ALT, AST and Alb), renal function (Scr, BUN and UA), serum sodium and potassium levels, HsCRP, RAAS activity (ACE, renin, Ang I, Ang II and ALD), dyslipidemia [TRIG, TC, LDL-C, ApoB, HDL-C, ApoA-I and Lp(a)], medical condition (T2D, EH, CAD(ACS), AF, and LCI), NYHA functional classification, and echocardiography index (RVD, RAD, LVD, LAD, and LVEF).

^b^**Model 4s**: It is the same as Model 4, and also including combined medication, such as antiplatelet drugs, warfarin, statins, RSIs, BBs, MRA, CCBs, diuretics, digoxin, nitrates, and hypoglycemic agents.

**Table S8. Association of *KATP* SNPs with new-onset HF risk in study participants.**

| ***KATP* SNPs** | | **HF (N/%)** | | ***χ2*** | ***P* value** | **Crude**  **OR (95% CI)** | **Crude**  ***P* value** | **Adjusted**  **OR (95% CI)^a^** | **Adjusted**  ***P* value^a^** | **Adjusted**  **OR (95% CI)^b^** | **Adjusted**  ***P* value^b^** | **Explained variance (%)^b^** |
| --- | --- | --- | --- | --- | --- | --- | --- | --- | --- | --- | --- | --- |
|  |  | **NO** | **YES** |  |  |  |  |  |  |  |  |  |
| *rs2285676* | *GG* | 342(34.6) | 110(39.0) | 1.864 | 0.174 | 1.00 |  | 1.00 |  | 1.00 |  |  |
|  | *AA+GA* | 646(65.4) | 172(61.0) |  |  | 0.84(0.66-1.07) | 0.152 | 0.91(0.70-1.18) | 0.468 | 0.94(0.71-1.23) | 0.631 | - |
| *rs11046182* | *GG* | 622(63.0) | 186(66.0) | 0.854 | 0.355 | 1.14(0.89-1.45) | 0.313 | 1.00(0.76-1.33) | 0.977 | 1.02(0.76-1.36) | 0.903 | - |
|  | *AA+GA* | 366(37.0) | 96(34.0) |  |  | 1.00 |  | 1.00 |  | 1.00 |  |  |
| *rs1799858* | *CC* | 668(67.6) | 132(46.8) | 40.726 | <0.001 | 1.00 |  | 1.00 |  | 1.00 |  |  |
|  | *TT+CT* | 320(32.4) | 150(53.2) |  |  | **2.12(1.68-2.68)** | **<0.001** | **2.65(1.99-3.51)** | **<0.001** | **2.78(2.07-3.74)** | **<0.001** | **3.48** |
| *rs4148671* | *CC* | 862(87.2) | 244(86.5) | 0.102 | 0.750 | 1.00 |  | 1.00 |  | 1.00 |  |  |
|  | *TT+CT* | 126(12.8) | 38(13.5) |  |  | 1.04(0.74-1.46) | 0.823 | 1.04(0.71-1.53) | 0.825 | 1.10(0.74-1.64) | 0.630 | - |
| *rs78148713* | *CC+CT* | 38(3.8) | 20(7.1) | 5.304 | 0.021 | 1.00 |  | 1.00 |  | 1.00 |  |  |
|  | *TT* | 950(96.2) | 262(92.9) |  |  | 0.57(0.36-0.89) | 0.014 | 0.67(0.38-1.17) | 0.159 | 0.63(0.35-1.13) | 0.122 | - |
| *rs145456027* | *CC+CT* | 24(2.4) | 12(4.3) | 2.656 | 0.103 | 1.00 |  | 1.00 |  | 1.00 |  |  |
|  | *TT* | 964(97.6) | 270(95.7) |  |  | 0.59(0.33-1.06) | 0.076 | 0.56(0.26-1.22) | 0.147 | 0.54(0.24-1.23) | 0.140 | - |
| *rs147265929* | *GG+GT* | 70(7.1) | 30(10.6) | 3.818 | 0.051 | 1.00 |  | 1.00 |  | 1.00 |  |  |
|  | *TT* | 918(92.9) | 252(89.4) |  |  | 0.69(0.47-1.01) | 0.055 | 0.67(0.43-1.06) | 0.088 | 0.69(0.43-1.10) | 0.121 | - |
| *rs61928479* | *AA* | 100(10.1) | 38(13.5) | 2.548 | 0.110 | 1.29(0.92-1.82) | 0.139 | 0.92(0.60-1.39) | 0.676 | 0.87(0.57-1.34) | 0.525 | - |
|  | *TT+AT* | 888(89.9) | 244(86.5) |  |  | 1.00 |  | 1.00 |  | 1.00 |  |  |
| *rs141294036* | *CC* | 380(38.5) | 104(36.9) | 0.233 | 0.629 | 1.00 |  | 1.00 |  | 1.00 |  |  |
|  | *TT+CT* | 608(61.5) | 178(63.1) |  |  | 1.06(0.83-1.35) | 0.658 | **1.38(1.02-1.86)** | **0.037** | **1.45(1.07-1.96)** | **0.015** | **0.46** |

^a^**Model 5**: After adjustment for gender, age, smoking, alcohol consumption, BMI, WBC, blood glucose levels (FBS, P2hBS and HbA1C), liver function (ALT, AST and Alb), renal function (Scr, BUN and UA), serum sodium and potassium levels, HsCRP, RAAS activity (ACE, renin, Ang I, Ang II and ALD), dyslipidemia [TRIG, TC, LDL-C, ApoB, HDL-C, ApoA-I and Lp(a)], medical condition (T2D, HTN, CAD(ACS), and AF), echocardiography index (RVD, RAD, LVD, LAD, and LVEF).

^b^**Model 5s**: It is the same as Model 5, and also including and also including combined medication, such as antiplatelet drugs, warfarin, statins, RSIs, BBs, MRA, CCBs, diuretics, digoxin, nitrates, and hypoglycemic agents.

**Table S9. Association of *KATP* SNPs with different types of HF in study participants.**

| ***KATP* SNPs** | **Subtypes of HF (N/%)** | **Genotypes (N/%)** | | **Crude**  **OR (95% CI)** | ***P* value** | **Adjusted**  **OR (95% CI)^a^** | ***P* value^a^** | **Adjusted**  **OR (95% CI)^b^** | ***P* value^b^** | **Explained variance (%)^b^** |
| --- | --- | --- | --- | --- | --- | --- | --- | --- | --- | --- |
|  |  | **CC (Ref.)** | **TT+CT** |  |  |  |  |  |  |  |
| *rs1799858* | Non-HF | 668(83.5) | 324(68.9) | Ref. | - | Ref. | - | Ref. | - | - |
|  | HFpEF | 84(10.5) | 98(20.9) | **2.41(1.75-3.31)** | **<0.001** | **3.33(2.24-4.95)** | **<0.001** | **3.46(2.31-5.18)** | **<0.001** | **2.78** |
|  | HFmrEF | 24(3.0) | 26(5.5) | 2.23(1.26-3.95) | 0.006 | 2.02(0.84-4.85) | 0.117 | 1.75(0.65-4.71) | 0.267 | - |
|  | HFrEF | 24(3.0) | 22(4.7) | 1.89(1.04-3.42) | 0.036 | 1.36(0.49-3.79) | 0.551 | 1.32(0.46-3.77) | 0.600 | - |
| *rs141294036* | Non-HF | 380(78.5) | 608(77.4) | Ref. | - | Ref. | - | Ref. | - | - |
|  | HFpEF | 78(16.1) | 108(13.7) | 0.83(0.60-1.14) | 0.248 | 0.83(0.56-1.23) | 0.347 | 0.88(0.60-1.31) | 0.528 | - |
|  | HFmrEF | 10(2.1) | 40(5.1) | **2.48(1.23-5.03)** | **0.011** | **2.70(1.06-6.85)** | **0.037** | **2.74(1.05-7.15)** | **0.039** | **0.33** |
|  | HFrEF | 16(3.3) | 30(3.8) | 1.16(0.63-2.16) | 0.631 | 1.04(0.38-2.87) | 0.941 | 1.11(0.39-3.20) | 0.846 | - |

^a^**Model 6**: After adjustment for gender, age, smoking, alcohol consumption, BMI, WBC, blood glucose levels (FBS, P2hBS and HbA1C), liver function (ALT, AST and Alb), renal function (Scr, BUN and UA), serum sodium and potassium levels, HsCRP, RAAS activity (ACE, renin, Ang I, Ang II and ALD), dyslipidemia [TRIG, TC, LDL-C, ApoB, HDL-C, ApoA-I and Lp(a)], medical condition (T2D, HTN, CAD(ACS), and AF), echocardiography index (RVD, RAD, LVD, LAD, and LVEF).

^b^**Model 6s**: It is the same as Model 6, and also including and also including combined medication, such as antiplatelet drugs, warfarin, statins, RSIs, BBs, MRA, CCBs, diuretics, digoxin, nitrates, and hypoglycemic agents.

**Table S10. Association of *KATP* SNPs with AF risk at enrollment in study participants.**

| ***KATP* SNPs** | | **AF at enrollment (N/%)** | | ***χ2*** | ***P* value** | **Crude**  **OR (95% CI)** | **Crude**  ***P* value** | **Adjusted**  **OR (95% CI)^a^** | **Adjusted**  ***P* value^a^** | **Adjusted**  **OR (95% CI)^b^** | **Adjusted**  ***P* value^b^** | **Explained variance (%)^b^** |
| --- | --- | --- | --- | --- | --- | --- | --- | --- | --- | --- | --- | --- |
|  |  | **NO** | **YES** |  |  |  |  |  |  |  |  |  |
| *rs2285676* | *GG* | 436(35.6) | 16(36.4) | 0.012 | 0.913 | 1.00 |  | 1.00 |  | 1.00 |  |  |
|  | *AA+GA* | 790(64.4) | 28(63.6) |  |  | 0.97(0.52-1.81) | 0.913 | 1.03(0.52-2.05) | 0.923 | 1.02(0.51-2.04) | 0.960 | - |
| *rs11046182* | *GG* | 774(63.1) | 34(77.3) | 3.670 | 0.055 | 1.99(0.97-4.06) | 0.060 | 1.98(0.90-4.34) | 0.088 | 1.95(0.88-4.31) | 0.100 | - |
|  | *AA+GA* | 452(36.9) | 10(22.7) |  |  | 1.00 |  | 1.00 |  | 1.00 |  |  |
| *rs1799858* | *CC* | 774(63.1) | 26(59.1) | 0.298 | 0.585 | 1.00 |  | 1.00 |  |  | 1.00 |  |
|  | *TT+CT* | 452(36.9) | 18(40.9) |  |  | 1.19(0.64-2.19) | 0.586 | **2.12(1.05-4.30)** | **0.037** | **2.15(1.06-4.39)** | **0.035** | **0.35** |
| *rs4148671* | *CC* | 1068(87.1) | 38(86.4) | 0.021 | 0.884 | 1.00 |  | 1.00 |  | 1.00 |  |  |
|  | *TT+CT* | 158(12.9) | 6(13.6) |  |  | 1.07(0.44-2.57) | 0.884 | 1.79(0.68-4.75) | 0.240 | 1.84(0.69-4.90) | 0.226 | - |
| *rs78148713* | *CC+CT* | 56(4.6) | 2(4.5) | 0.000 | 0.994 | 1.00 |  | 1.00 |  | 1.00 |  |  |
|  | *TT* | 1170(95.4) | 42(95.5) |  |  | 1.01(0.24-4.26) | 0.994 | 1.20(0.24-5.93) | 0.819 | 1.15(0.23-5.76) | 0.866 | - |
| *rs145456027* | *CC+CT* | 36(2.9) | 0(0.0) | 1.330 | 0.249 | - |  | - |  | - |  |  |
|  | *TT* | 1190(97.1) | 44(100.0) |  |  | - | - | - | - | - | - | - |
| *rs147265929* | *GG+GT* | 96(7.8) | 4(9.1) | 0.093 | 0.760 | 1.00 |  | 1.00 |  | 1.00 |  |  |
|  | *TT* | 1130(92.2) | 40(90.9) |  |  | 0.85(0.30-2.43) | 0.761 | 1.30(0.37-4.58) | 0.658 | 1.21(0.34-4.40) | 0.769 | - |
| *rs61928479* | *AA* | 134(10.9) | 4(9.1) | 0.148 | 0.700 | 0.82(0.29-2.31) | 0.701 | 0.80(0.25-2.56) | 0.701 | 0.83(0.26-2.67) | 0.753 | - |
|  | *TT+AT* | 1092(89.1) | 40(90.9) |  |  | 1.00 |  | 1.00 |  | 1.00 |  |  |
| *rs141294036* | *CC* | 458(37.8) | 26(44.8) | 1.163 | 0.281 | **2.00(1.09-3.66)** | **0.025** | **2.39(1.15-4.96)** | **0.019** | **2.42(1.15-5.07)** | **0.020** | **0.43** |
|  | *TT+CT* | 754(62.2) | 32(55.2) |  |  | 1.00 |  | 1.00 |  | 1.00 |  |  |

^a^**Model 7**: After adjustment for gender, age, smoking, alcohol consumption, BMI, WBC, blood glucose levels (FBS, P2hBS and HbA1C), liver function (ALT, AST and Alb), renal function (Scr, BUN and UA), serum sodium and potassium levels, HsCRP, RAAS activity (ACE, renin, Ang I, Ang II and ALD), dyslipidemia [TRIG, TC, LDL-C, ApoB, HDL-C, ApoA-I and Lp(a)], medical condition (T2D, HTN, CAD(ACS), and HF), echocardiography index (RVD, RAD, LVD, LAD, and LVEF).

^b^**Model 7s**: It is the same as Model 7, and also including and also including combined medication, such as antiplatelet drugs, warfarin, statins, RSIs, BBs, MRA, CCBs, diuretics, digoxin, nitrates, and hypoglycemic agents.

**Table S11. Association of *KATP* SNPs with new-onset AF risk in study participants.**

| ***KATP* SNPs** | | **New-onset AF**  **(N/%)** | | ***χ2*** | ***P* value** | **Crude**  **OR (95% CI)** | **Crude**  ***P* value** | **Adjusted**  **OR (95% CI)^a^** | **Adjusted**  ***P* value^a^** | **Adjusted**  **OR (95% CI)^b^** | **Adjusted**  ***P* value^b^** | **Explained variance (%)^b^** |
| --- | --- | --- | --- | --- | --- | --- | --- | --- | --- | --- | --- | --- |
|  |  | **NO** | **YES** |  |  |  |  |  |  |  |  |  |
| *rs2285676* | *GG* | 408(36.0) | 28(29.8) | 1.482 | 0.223 | 1.00 |  | 1.00 |  | 1.00 |  |  |
|  | *AA+GA* | 724(64.0) | 66(70.2) |  |  | 1.31(0.84-2.04) | 0.233 | 1.35(0.83-2.20) | 0.222 | 1.46(0.88-2.40) | 0.141 | - |
| *rs11046182* | *GG* | 724(64.0) | 50(53.2) | 4.322 | 0.038 | 0.65(0.43-0.98) | 0.038 | 0.85(0.53-1.37) | 0.508 | 0.76(0.48-1.23) | 0.267 | - |
|  | *AA+GA* | 408(36.0) | 44(46.8) |  |  | 1.00 |  | 1.00 |  | 1.00 |  |  |
| *rs1799858* | *CC* | 730(64.5) | 44(46.8) | 11.655 | 0.001 | 1.00 |  | 1.00 |  | 1.00 |  |  |
|  | *TT+CT* | 402(35.5) | 50(53.2) |  |  | **2.00(1.33-3.00)** | **0.001** | **2.09(1.29-3.38)** | **0.003** | **2.05(1.25-3.37)** | **0.004** | **0.66** |
| *rs4148671* | *CC* | 982(86.7) | 86(91.5) | 1.737 | 0.187 | 1.00 |  | 1.00 |  | 1.00 |  |  |
|  | *TT+CT* | 150(13.3) | 8(8.5) |  |  | 0.62(0.30-1.28) | 0.195 | 0.56(0.24-1.31) | 0.182 | 0.50(0.21-1.21) | 0.123 | - |
| *rs78148713* | *CC+CT* | 52(4.6) | 4(4.3) | 0.023 | 0.880 | 1.00 |  | 1.00 |  | 1.00 |  |  |
|  | *TT* | 1080(95.4) | 90(95.7) |  |  | 1.09(0.40-2.96) | 0.869 | 1.30(0.43-3.95) | 0.649 | 1.61(0.52-5.04) | 0.412 | - |
| *rs145456027* | *CC+CT* | 32(2.8) | 4(4.3) | 0.621 | 0.431 | 1.00 |  | 1.00 |  | 1.00 |  |  |
|  | *TT* | 1100(97.2) | 90(95.7) |  |  | 0.67(0.25-1.82) | 0.432 | 0.71(0.23-2.71) | 0.548 | 0.73(0.23-2.30) | 0.593 | - |
| *rs147265929* | *GG+GT* | 82(7.2) | 14(14.9) | 7.037 | 0.008 | 1.00 |  | 1.00 |  | 1.00 |  |  |
|  | *TT* | 1050(92.8) | 80(85.1) |  |  | 0.46(0.26-0.82) | 0.008 | 0.52(0.25-1.06) | 0.072 | 0.56(0.27-1.19) | 0.132 | - |
| *rs61928479* | *AA* | 114(10.1) | 20(21.3) | 11.195 | 0.001 | 2.21(1.35-3.63) | 0.002 | 1.26(0.64-2.47) | 0.510 | 1.18(0.58-2.41) | 0.655 | - |
|  | *TT+AT* | 1018(89.9) | 74(78.7) |  |  | 1.00 |  | 1.00 |  | 1.00 |  |  |
| *rs141294036* | *CC* | 410(36.2) | 50(53.2) | 10.665 | 0.001 | **1.93(1.29-2.89)** | **0.001** | **2.42(1.50-3.90)** | **<0.001** | **2.31(1.40-3.82)** | **0.001** | **0.86** |
|  | *TT+CT* | 722(63.8) | 44(46.8) |  |  | 1.00 |  | 1.00 |  | 1.00 |  |  |

^a^**Model 8**: After adjustment for gender, age, smoking, alcohol consumption, BMI, WBC, blood glucose levels (FBS, P2hBS and HbA1C), liver function (ALT, AST and Alb), renal function (Scr, BUN and UA), serum sodium and potassium levels, HsCRP, RAAS activity (ACE, renin, Ang I, Ang II and ALD), dyslipidemia [TRIG, TC, LDL-C, ApoB, HDL-C, ApoA-I and Lp(a)], medical condition (T2D, HTN, CAD(ACS), HF), echocardiography index (RVD, RAD, LVD, LAD, and LVEF).

^b^**Model 8s**: It is the same as Model 8, and also including and also including combined medication, such as antiplatelet drugs, warfarin, statins, RSIs, BBs, MRA, CCBs, diuretics, digoxin, nitrates, and hypoglycemic agents.

**Table S12. Association of *KATP* SNPs with total AF risk in study participants.**

| ***KATP* SNPs** | | **Total AF**  **(N/%)** | | ***χ2*** | ***P* value** | **Crude**  **OR (95% CI)** | **Crude**  ***P* value** | **Adjusted**  **OR (95% CI)^a^** | **Adjusted**  ***P* value^a^** | **Adjusted**  **OR (95% CI)^b^** | **Adjusted**  ***P* value^b^** | **Explained variance (%)^b^** |
| --- | --- | --- | --- | --- | --- | --- | --- | --- | --- | --- | --- | --- |
|  |  | **NO** | **YES** |  |  |  |  |  |  |  |  |  |
| *rs2285676* | *GG* | 408(36.0) | 44(31.9) | 0.928 | 0.335 | 1.00 |  | 1.00 |  | 1.00 |  |  |
|  | *AA+GA* | 724(64.0) | 94(68.1) |  |  | 1.20(0.83-1.76) | 0.336 | 1.24(0.81-1.89) | 0.321 | 1.29(0.83-2.00) | 0.256 | - |
| *rs11046182* | *GG* | 724(64.0) | 84(60.9) | 0.507 | 0.477 | 0.88(0.61-1.26) | 0.477 | 0.78(0.52-1.19) | 0.246 | 0.69(0.45-1.07) | 0.095 | - |
|  | *AA+GA* | 408(36.0) | 54(39.1) |  |  | 1.00 |  | 1.00 |  | 1.00 |  |  |
| *rs1799858* | *CC* | 730(64.5) | 70(50.7) | 9.995 | 0.002 | 1.00 |  | 1.00 |  | 1.00 |  |  |
|  | *TT+CT* | 402(35.5) | 68(49.3) |  |  | **1.76(1.24-2.52)** | **0.002** | **1.84(1.21-2.81)** | **0.005** | **2.00(1.28-3.11)** | **0.002** | **0.73** |
| *rs4148671* | *CC* | 982(86.7) | 124(89.9) | 1.055 | 0.304 | 1.00 |  | 1.00 |  | 1.00 |  |  |
|  | *TT+CT* | 150(13.3) | 14(10.1) |  |  | 0.74(0.41-1.32) | 0.306 | 0.76(0.41-1.44) | 0.405 | 0.62(0.32-1.23) | 0.171 | - |
| *rs78148713* | *CC+CT* | 52(4.6) | 6(4.3) | 0.017 | 0.896 | 1.00 |  | 1.00 |  | 1.00 |  |  |
|  | *TT* | 1080(95.4) | 132(95.7) |  |  | 1.06(0.45-2.51) | 0.896 | 1.20(0.46-3.14) | 0.707 | 1.48(0.54-4.05) | 0.441 | - |
| *rs145456027* | *CC+CT* | 32(2.8) | 4(2.9) | 0.621 | 0.431 | 1.00 |  | 1.00 |  | 1.00 |  |  |
|  | *TT* | 1100(97.2) | 134(97.1) |  |  | 0.98(0.34-2.80) | 0.962 | 1.17(0.37-3.72) | 0.795 | 1.25(0.37-4.20) | 0.724 | - |
| *rs147265929* | *GG+GT* | 82(7.2) | 18(13.0) | 7.037 | 0.008 | 1.00 |  | 1.00 |  | 1.00 |  |  |
|  | *TT* | 1050(92.8) | 120(87.0) |  |  | 0.52(0.30-0.90) | 0.019 | 0.57(0.30-1.08) | 0.082 | 0.57(0.29-1.11) | 0.099 | - |
| *rs61928479* | *AA* | 114(10.1) | 24(17.4) | 6.806 | 0.009 | 1.88(1.16-3.04) | 0.010 | 1.27(0.71-2.25) | 0.422 | 1.04(0.56-1.91) | 0.910 | - |
|  | *TT+AT* | 1018(89.9) | 114(82.6) |  |  | 1.00 |  | 1.00 |  | 1.00 |  |  |
| *rs141294036* | *CC* | 410(36.2) | 74(53.6) | 15.797 | <0.001 | **2.04(1.43-2.91)** | **<0.001** | **2.29(1.49-3.50)** | **<0.001** | **2.38(1.52-3.73)** | **<0.001** | **1.12** |
|  | *TT+CT* | 722(63.8) | 64(46.4) |  |  | 1.00 |  | 1.00 |  | 1.00 |  |  |

^a^**Model 9**: After adjustment for gender, age, smoking, alcohol consumption, BMI, WBC, blood glucose levels (FBS, P2hBS and HbA1C), liver function (ALT, AST and Alb), renal function (Scr, BUN and UA), serum sodium and potassium levels, HsCRP, RAAS activity (ACE, renin, Ang I, Ang II and ALD), dyslipidemia [TRIG, TC, LDL-C, ApoB, HDL-C, ApoA-I and Lp(a)], medical condition (T2D, HTN, CAD(ACS) and HF), echocardiography index (RVD, RAD, LVD, LAD, and LVEF).

^b^**Model 9s**: It is the same as Model 9, and also including and also including combined medication, such as antiplatelet drugs, warfarin, statins, RSIs, BBs, MRA, CCBs, diuretics, digoxin, nitrates, and hypoglycemic agents.

**Table S13. Association of *KATP* SNPs with LCI risk at enrollment in study participants.**

| ***KATP* SNPs** | | **LCI (N/%)** | | ***χ2*** | ***P* value** | **Crude**  **OR (95% CI)** | **Crude**  ***P* value** | **Adjusted**  **OR (95% CI)^a^** | **Adjusted**  ***P* value^a^** | **Adjusted**  **OR (95% CI)^b^** | **Adjusted**  ***P* value^b^** | **Explained variance (%)^b^** |
| --- | --- | --- | --- | --- | --- | --- | --- | --- | --- | --- | --- | --- |
|  |  | **NO** | **YES** |  |  |  |  |  |  |  |  |  |
| *rs2285676* | *GG* | 434(35.6) | 18(34.6) | 0.022 | 0.881 | 1.00 |  | 1.00 |  | 1.00 |  |  |
|  | *AA+GA* | 784(64.4) | 34(65.4) |  |  | 1.05(0.58-1.87) | 0.881 | 0.90(0.48-1.69) | 0.739 | 0.85(0.45-1.60) | 0.607 | - |
| *rs11046182* | *GG* | 768(63.1) | 40(76.9) | 4.145 | 0.042 | 1.95(1.01-3.76) | 0.045 | 1.94(0.93-4.05) | 0.079 | 2.00(0.95-4.21) | 0.068 | - |
|  | *AA+GA* | 450(36.9) | 12(23.1) |  |  | 1.00 |  | 1.00 |  | 1.00 |  |  |
| *rs1799858* | *CC* | 760(62.4) | 40(76.9) | 4.514 | 0.034 | **2.01(1.04-3.87)** | **0.037** | **2.22(1.08-4.56)** | **0.029** | **2.27(1.10-4.71)** | **0.027** | **0.38** |
|  | *TT+CT* | 458(37.6) | 12(23.1) |  |  | 1.00 |  | 1.00 |  | 1.00 |  |  |
| *rs4148671* | *CC* | 1060(87.0) | 46(88.5) | 0.091 | 0.763 | 1.00 |  | 1.00 |  | 1.00 |  |  |
|  | *TT+CT* | 158(13.0) | 6(11.5) |  |  | 0.88(0.37-2.08) | 0.763 | 1.06(0.42-2.67) | 0.903 | 1.13(0.45-2.84) | 0.795 | - |
| *rs78148713* | *CC+CT* | 56(4.6) | 2(3.8) | 0.065 | 0.799 | 1.00 |  | 1.00 |  | 1.00 |  |  |
|  | *TT* | 1162(95.4) | 50(96.2) |  |  | 1.21(0.29-5.08) | 0.800 | 1.25(0.28-5.69) | 0.770 | 1.38(0.30-6.34) | 0.683 | - |
| *rs145456027* | *CC+CT* | 36(3.0) | 0(0.0) | 1.773 | 0.183 | - |  | - |  | - |  |  |
|  | *TT* | 1182(97.0) | 52(100.0) |  |  | - | - | - | - | - | - | - |
| *rs147265929* | *GG+GT* | 96(7.9) | 4(7.7) | 0.002 | 0.960 | 1.00 |  | 1.00 |  | 1.00 |  |  |
|  | *TT* | 1122(92.1) | 48(92.3) |  |  | 1.03(0.36-2.91) | 0.960 | 0.88(0.30-2.65) | 0.826 | 0.92(0.30-2.80) | 0.884 | - |
| *rs61928479* | *AA* | 130(10.7) | 8(15.4) | 1.143 | 0.285 | 1.52(0.70-3.30) | 0.288 | 1.40(0.60-3.28) | 0.443 | 1.33(0.56-3.16) | 0.518 | - |
|  | *TT+AT* | 1088(89.3) | 44(84.6) |  |  | 1.00 |  | 1.00 |  | 1.00 |  |  |
| *rs141294036* | *CC* | 464(38.1) | 20(38.5) | 0.003 | 0.958 | 1.02(0.57-1.80) | 0.958 | 1.33(0.70-2.52) | 0.389 | 1.31(0.69-2.51) | 0.409 | - |
|  | *TT+CT* | 754(61.9) | 32(61.5) |  |  | 1.00 |  | 1.00 |  | 1.00 |  |  |

^a^**Model 10**: After adjustment for gender, age, smoking, alcohol consumption, BMI, WBC, T2D, SBP, DBP, blood glucose levels (FBS, P2hBS and HbA1C), liver function (ALT, AST and Alb), renal function (Scr, BUN and UA), serum sodium and potassium levels, HsCRP, and dyslipidemia [TRIG, TC, LDL-C, ApoB, HDL-C, ApoA-I and Lp(a)].

^b^**Model 10s**: It is the same as Model 10, and also including RAAS activity (ACE, renin, Ang I, Ang II and ALD).

**Table S14. Association of *KATP* SNPs with increased serum TRIG level (≥ 1.70 mmol/L) in study participants.**

| ***KATP* SNPs** | | **TRIG ≥ 1.70 mmol/L (N/%)** | | ***χ2*** | ***P* value** | **Crude**  **OR (95% CI)** | **Crude**  ***P* value** | **Adjusted**  **OR (95% CI)^a^** | **Adjusted**  ***P* value^a^** | **Adjusted**  **OR (95% CI)^b^** | **Adjusted**  ***P* value^b^** | **Explained variance (%)^b^** |
| --- | --- | --- | --- | --- | --- | --- | --- | --- | --- | --- | --- | --- |
|  |  | **NO** | **YES** |  |  |  |  |  |  |  |  |  |
| *rs2285676* | *GG* | 314(38.4) | 138(30.5) | 7.837 | 0.005 | 1.00 |  | 1.00 |  | 1.00 |  |  |
|  | *AA+GA* | 504(61.6) | 314(69.5) |  |  | **1.42(1.11-1.81)** | **0.005** | **1.35(1.03-1.77)** | **0.028** | **1.37(1.02-1.84)** | **0.035** | **0.35** |
| *rs11046182* | *GG* | 532(65.0) | 276(61.1) | 1.987 | 0.159 | 0.81(0.63-1.03) | 0.090 | 0.82(0.62-1.04) | 0.099 | 0.76(0.57-1.02) | 0.067 | - |
|  | *AA+GA* | 286(35.0) | 176(38.9) |  |  | 1.00 |  | 1.00 |  | 1.00 |  |  |
| *rs1799858* | *CC* | 508(62.1) | 292(64.6) | 0.780 | 0.377 | 1.21(0.95-1.55) | 0.124 | 1.25(0.95-1.65) | 0.109 | 1.19(0.88-1.59) | 0.262 | - |
|  | *TT+CT* | 310(37.9) | 160(35.4) |  |  | 1.00 |  | 1.00 |  | 1.00 |  |  |
| *rs4148671* | *CC* | 728(89.0) | 378(83.6) | 7.463 | 0.006 | 1.00 |  | 1.00 |  | 1.00 |  |  |
|  | *TT+CT* | 90(11.0) | 74(16.4) |  |  | 1.73(1.17-2.55) | 0.006 | 1.97(1.28-3.03) | 0.002 | 1.62(1.01-2.60) | 0.045 | - |
| *rs78148713* | *CC+CT* | 40(4.9) | 18(4.0) | 0.550 | 0.458 | 1.00 |  | 1.00 |  | 1.00 |  |  |
|  | *TT* | 778(95.1) | 434(96.0) |  |  | 1.24(0.70-2.19) | 0.459 | 1.10(0.61-2.00) | 0.746 | 1.88(0.85-4.16) | 0.118 | - |
| *rs145456027* | *CC+CT* | 20(2.4) | 16(3.5) | 1.267 | 0.260 | 1.00 |  | 1.00 |  | 1.00 |  |  |
|  | *TT* | 798(97.6) | 436(96.5) |  |  | 0.57(0.26-1.23) | 0.152 | 0.61(0.27-1.40) | 0.245 | 0.65(0.25-1.66) | 0.365 | - |
| *rs147265929* | *GG+GT* | 58(7.1) | 42(9.3) | 1.945 | 0.163 | 1.00 |  | 1.00 |  | 1.00 |  |  |
|  | *TT* | 760(92.9) | 410(90.7) |  |  | 0.75(0.49-1.16) | 0.196 | 0.64(0.39-1.04) | 0.071 | 0.61(0.36-1.02) | 0.061 | - |
| *rs61928479* | *AA* | 72(8.8) | 66(14.6) | 10.111 | 0.001 | 1.60(1.10-2.34) | 0.015 | 1.30(0.86-1.98) | 0.216 | 1.36(0.84-2.15) | 0.182 | - |
|  | *TT+AT* | 746(91.2) | 386(85.4) |  |  | 1.00 |  | 1.00 |  | 1.00 |  |  |
| *rs141294036* | *CC* | 318(38.9) | 166(36.7) | 0.570 | 0.450 | 0.99(0.77-1.26) | 0.925 | 0.88(0.67-1.15) | 0.344 | 0.84(0.62-1.14) | 0.261 | - |
|  | *TT+CT* | 500(61.1) | 286(63.3) |  |  | 1.00 |  | 1.00 |  | 1.00 |  |  |

^a^**Model 11**: After adjustment for gender, age, smoking, alcohol consumption, BMI, WBC, T2D,SBP, DBP, blood glucose levels (FBS, P2hBS and HbA1C), liver function (ALT, AST and Alb), renal function (Scr, BUN and UA), serum sodium and potassium levels, HsCRP, and RAAS activity (ACE, renin, Ang I, Ang II and ALD).

^b^**Model 11a**: It is the same as Model 11, and also including dyslipidemia, such as TC, LDL-C, ApoB, HDL-C, ApoA-I and Lp(a).

**Table S15. Association of *KATP* SNPs with increased serum LDL-C level (≥ 1.40 mmol/L) in study participants.**

| ***KATP* SNPs** | | **LDL-C ≥ 1.40 mmol/L (N/%)** | | ***χ2*** | ***P* value** | **Crude**  **OR (95% CI)** | **Crude**  ***P* value** | **Adjusted**  **OR (95% CI)^a^** | **Adjusted**  ***P* value^a^** | **Adjusted**  **OR (95% CI)^b^** | **Adjusted**  ***P* value^b^** | **Explained variance (%)^b^** |
| --- | --- | --- | --- | --- | --- | --- | --- | --- | --- | --- | --- | --- |
|  |  | **NO** | **YES** |  |  |  |  |  |  |  |  |  |
| *rs2285676* | *GG* | 60(45.5) | 392(34.4) | 6.253 | 0.012 | 1.00 |  | 1.00 |  | 1.00 |  |  |
|  | *AA+GA* | 72(54.5) | 746(65.6) |  |  | **1.59(1.10-2.28)** | **0.013** | **1.82(1.22-2.71)** | **0.003** | **1.82(1.13-2.92)** | **0.014** | **0.47** |
| *rs11046182* | *GG* | 82(62.1) | 726(63.8) | 0.143 | 0.705 | 1.07(0.74-1.56) | 0.705 | 0.99(0.67-1.48) | 0.969 | 1.25(0.79-1.97) | 0.348 | - |
|  | *AA+GA* | 50(37.9) | 412(36.2) |  |  | 1.00 |  | 1.00 |  | 1.00 |  |  |
| *rs1799858* | *CC* | 98(74.2) | 702(61.7) | 7.998 | 0.005 | 1.00 |  | 1.00 |  | 1.00 |  |  |
|  | *TT+CT* | 34(25.8) | 436(38.3) |  |  | **1.79(1.19-2.69)** | **0.005** | **1.88(1.22-2.91)** | **0.005** | **2.26(1.39-3.69)** | **0.001** | **0.84** |
| *rs4148671* | *CC* | 112(84.8) | 994(87.3) | 0.656 | 0.418 | 1.00 |  | 1.00 |  | 1.00 |  |  |
|  | *TT+CT* | 20(15.2) | 144(12.7) |  |  | 0.81(0.49-1.35) | 0.419 | 0.80(0.46-1.40) | 0.437 | 0.69(0.36-1.34) | 0.275 | - |
| *rs78148713* | *CC+CT* | 6(4.5) | 52(4.6) | <0.001 | 0.990 | 1.00 |  | 1.00 |  | 1.00 |  |  |
|  | *TT* | 126(95.5) | 1086(95.4) |  |  | 1.00(0.42-2.36) | 0.990 | 0.92(0.38-2.27) | 0.863 | 0.61(0.22-1.67) | 0.331 | - |
| *rs145456027* | *CC+CT* | 2(1.5) | 34(3.0) | 0.931 | 0.335 | 1.00 |  | 1.00 |  | 1.00 |  |  |
|  | *TT* | 120(98.5) | 1104(97.0) |  |  | 0.50(0.12-2.10) | 0.344 | 0.55(0.13-2.41) | 0.429 | 0.35(0.07-1.83) | 0.214 | - |
| *rs147265929* | *GG+GT* | 6(4.5) | 94(8.3) | 2.250 | 0.134 | 1.00 |  | 1.00 |  | 1.00 |  |  |
|  | *TT* | 126(95.5) | 1044(91.7) |  |  | 0.53(0.23-1.23) | 0.140 | 0.49(0.20-1.20) | 0.120 | 0.48(0.18-1.31) | 0.153 | - |
| *rs61928479* | *AA* | 12(9.1) | 126(11.1) | 0.479 | 0.489 | 1.25(0.67-2.32) | 0.490 | 1.59(0.76-3.33) | 0.224 | 1.33(0.60-2.97) | 0.488 | - |
|  | *TT+AT* | 120(90.9) | 1012(88.9) |  |  | 1.00 |  | 1.00 |  | 1.00 |  |  |
| *rs141294036* | *CC* | 50(37.9) | 434(38.1) | 0.003 | 0.954 | 1.01(0.70-1.47) | 0.954 | 1.01(0.67-1.50) | 0.978 | 1.10(0.69-1.74) | 0.696 | - |
|  | *TT+CT* | 82(62.1) | 704(61.9) |  |  | 1.00 |  | 1.00 |  | 1.00 |  |  |

^a^Model 11.

^b^**Model 11b**: It is the same as Model 11, and also including dyslipidemia, such as TRIG, TC, ApoB, HDL-C, ApoA-I and Lp(a).

**Table S16. Association of *KATP* SNPs with increased serum ApoB level (≥ 80 mg/dL) in study participants.**

| ***KATP* SNPs** | | **ApoB ≥ 80 mg/dL (N/%)** | | ***χ2*** | ***P* value** | **Crude**  **OR (95% CI)** | **Crude**  ***P* value** | **Adjusted**  **OR (95% CI)^a^** | **Adjusted**  ***P* value^a^** | **Adjusted**  **OR (95% CI)^b^** | **Adjusted**  ***P* value^b^** | **Explained variance (%)^b^** |
| --- | --- | --- | --- | --- | --- | --- | --- | --- | --- | --- | --- | --- |
|  |  | **NO** | **YES** |  |  |  |  |  |  |  |  |  |
| *rs2285676* | *GG* | 222(38.8) | 230(33.0) | 4.709 | 0.030 | 1.00 |  | 1.00 |  | 1.00 |  |  |
|  | *AA+GA* | 350(61.2) | 468(67.0) |  |  | **1.29(1.03-1.63)** | **0.030** | **1.41(1.09-1.81)** | **0.008** | **1.34(1.03-1.74)** | **0.031** | **0.37** |
| *rs11046182* | *GG* | 384(67.1) | 424(60.7) | 5.543 | 0.019 | 0.76(0.60-0.96) | 0.019 | 0.80(0.60-1.07) | 0.134 | 0.85(0.62-1.17) | 0.320 | - |
|  | *AA+GA* | 188(32.9) | 274(39.3) |  |  | 1.00 |  | 1.00 |  | 1.00 |  |  |
| *rs1799858* | *CC* | 356(62.2) | 444(63.6) | 0.254 | 0.614 | 1.06(0.84-1.33) | 0.614 | 1.16(0.90-1.49) | 0.243 | 1.18(0.91-1.52) | 0.226 | - |
|  | *TT+CT* | 216(37.8) | 254(36.4) |  |  | 1.00 |  | 1.00 |  | 1.00 |  |  |
| *rs4148671* | *CC* | 500(87.4) | 606(86.8) | 0.098 | 0.754 | 1.00 |  | 1.00 |  | 1.00 |  |  |
|  | *TT+CT* | 72(12.6) | 92(13.2) |  |  | 1.05(0.76-1.47) | 0.754 | 1.10(0.77-1.57) | 0.593 | 1.10(0.74-1.64) | 0.625 | - |
| *rs78148713* | *CC+CT* | 34(5.9) | 24(3.4) | 4.529 | 0.033 | 1.00 |  | 1.00 |  | 1.00 |  |  |
|  | *TT* | 538(94.1) | 674(96.6) |  |  | 1.78(1.04-3.03) | 0.035 | 1.76(0.96-3.23) | 0.069 | 1.43(0.75-2.72) | 0.281 | - |
| *rs145456027* | *CC+CT* | 14(2.4) | 22(3.2) | 0.566 | 0.452 | 1.00 |  | 1.00 |  | 1.00 |  |  |
|  | *TT* | 558(97.6) | 676(96.8) |  |  | 0.77(0.39-1.52) | 0.453 | 1.13(0.55-2.30) | 0.740 | 1.12(0.53-2.40) | 0.763 | - |
| *rs147265929* | *GG+GT* | 40(7.0) | 60(8.6) | 1.114 | 0.291 | 1.00 |  | 1.00 |  | 1.00 |  |  |
|  | *TT* | 532(93.0) | 638(91.4) |  |  | 0.80(0.53-1.21) | 0.292 | 0.73(0.46-1.14) | 0.164 | 0.83(0.50-1.35) | 0.448 | - |
| *rs61928479* | *AA* | 64(11.2) | 74(10.6) | 0.112 | 0.738 | 0.94(0.66-1.34) | 0.738 | 0.98(0.66-1.44) | 0.908 | 0.86(0.56-1.32) | 0.487 | - |
|  | *TT+AT* | 508(88.8) | 624(89.4) |  |  | 1.00 |  | 1.00 |  | 1.00 |  |  |
| *rs141294036* | *CC* | 200(35.0) | 284(40.7) | 4.365 | 0.037 | 1.28(1.02-1.60) | 0.037 | 1.23(0.96-1.59) | 0.106 | 1.21(0.91-1.60) | 0.190 | - |
|  | *TT+CT* | 372(65.0) | 414(59.3) |  |  | 1.00 |  | 1.00 |  | 1.00 |  |  |

^a^Model 11.

^b^**Model 11c**: It is the same as Model 11, and also including dyslipidemia, such as TRIG, TC, LDL-C, HDL-C, ApoA-I and Lp(a).

**Table S17. Association of *KATP* SNPs with decreased serum ApoA-I level (< 120 mg/dL) in study participants.**

| ***KATP* SNPs** | | **ApoA-I < 120 mg/dL (N/%)** | | ***χ2*** | ***P* value** | **Crude**  **OR (95% CI)** | **Crude**  ***P* value** | **Adjusted**  **OR (95% CI)^a^** | **Adjusted**  ***P* value^a^** | **Adjusted**  **OR (95% CI)^b^** | **Adjusted**  ***P* value^b^** | **Explained variance (%)^b^** |
| --- | --- | --- | --- | --- | --- | --- | --- | --- | --- | --- | --- | --- |
|  |  | **NO** | **YES** |  |  |  |  |  |  |  |  |  |
| *rs2285676* | *GG* | 118(32.4) | 334(36.9) | 2.241 | 0.134 | 1.00 |  | 1.00 |  | 1.00 |  |  |
|  | *AA+GA* | 246(67.6) | 572(63.1) |  |  | 0.82(0.64-1.06) | 0.135 | 0.85(0.64-1.13) | 0.260 | 0.78(0.58-1.05) | 0.101 | - |
| *rs11046182* | *GG* | 234(64.3) | 574(63.4) | 0.097 | 0.755 | 0.96(0.75-1.24) | 0.755 | 0.99(0.75-1.31) | 0.923 | 1.07(0.78-1.46) | 0.682 | - |
|  | *AA+GA* | 130(35.7) | 332(36.6) |  |  | 1.00 |  | 1.00 |  | 1.00 |  |  |
| *rs1799858* | *CC* | 250(68.7) | 550(60.7) | 7.084 | 0.008 | 1.00 |  | 1.00 |  | 1.00 |  |  |
|  | *TT+CT* | 114(31.3) | 356(39.3) |  |  | **1.42(1.10-1.84)** | **0.008** | **1.42(1.05-1.92)** | **0.022** | **1.57(1.13-2.19)** | **0.008** | **0.55** |
| *rs4148671* | *CC* | 328(90.1) | 778(85.9) | 4.147 | 0.042 | 1.00 |  | 1.00 |  | 1.00 |  |  |
|  | *TT+CT* | 36(9.9) | 128(14.1) |  |  | 1.50(1.01-2.22) | 0.043 | 1.39(0.86-2.23) | 0.176 | 1.60(0.93-2.73) | 0.087 | - |
| *rs78148713* | *CC+CT* | 12(3.3) | 46(5.1) | 1.889 | 0.169 | 1.00 |  | 1.00 |  | 1.00 |  |  |
|  | *TT* | 352(96.7) | 860(94.9) |  |  | 0.64(0.33-1.22) | 0.173 | 0.62(0.31-1.22) | 0.168 | 0.80(0.38-1.69) | 0.555 | - |
| *rs145456027* | *CC+CT* | 8(2.2) | 28(3.1) | 0.751 | 0.386 | 1.00 |  | 1.00 |  | 1.00 |  |  |
|  | *TT* | 356(97.8) | 878(96.9) |  |  | 0.75(0.32-1.56) | 0.388 | 0.52(0.22-1.20) | 0.126 | 0.66(0.26-1.68) | 0.380 | - |
| *rs147265929* | *GG+GT* | 24(6.6) | 76(8.4) | 1.154 | 0.283 | 1.00 |  | 1.00 |  | 1.00 |  |  |
|  | *TT* | 340(93.4) | 830(91.6) |  |  | 0.77(0.48-1.24) | 0.284 | 0.76(0.45-1.29) | 0.315 | 0.68(0.38-1.23) | 0.203 | - |
| *rs61928479* | *AA* | 24(6.6) | 114 (12.6) | 9.618 | 0.002 | 2.04(1.29-3.22) | 0.002 | 1.98(1.20-3.28) | 0.008 | 2.05(1.22-3.46) | 0.007 | - |
|  | *TT+AT* | 340(93.4) | 792(87.4) |  |  | 1.00 |  | 1.00 |  | 1.00 |  |  |
| *rs141294036* | *CC* | 156(42.9) | 328(36.2) | 4.875 | 0.027 | 1.00 |  | 1.00 |  | 1.00 |  |  |
|  | *TT+CT* | 208(57.1) | 578(63.8) |  |  | **1.32(1.03-1.69)** | **0.027** | **1.51(1.14-1.99)** | **0.004** | **1.72(1.28-2.30)** | <**0.001** | **1.03** |

^a^ Model 11.

^b^**Model 11d**: It is the same as Model 11, and also including dyslipidemia, such as TRIG, TC, LDL-C, ApoB, HDL-C, and Lp(a).

**Table S18. Association of *KATP* SNPs with increased serum Lp(a) level (≥ 300 mg/dL) in study participants.**

| ***KATP* SNPs** | | **Lp(a) ≥ 300 mg/dL (N/%)** | | ***χ2*** | ***P* value** | **Crude**  **OR (95% CI)** | **Crude**  ***P* value** | **Adjusted**  **OR (95% CI)^a^** | **Adjusted**  ***P* value^a^** | **Adjusted**  **OR (95% CI)^b^** | **Adjusted**  ***P* value^b^** | **Explained variance (%)^b^** |
| --- | --- | --- | --- | --- | --- | --- | --- | --- | --- | --- | --- | --- |
|  |  | **NO** | **YES** |  |  |  |  |  |  |  |  |  |
| *rs2285676* | *GG* | 330(38.1) | 122(30.2) | 7.516 | 0.006 | 1.00 |  | 1.00 |  | 1.00 |  |  |
|  | *AA+GA* | 536(61.9) | 282(69.8) |  |  | **1.42(1.11-1.83)** | **0.006** | **1.48(1.13-1.92)** | **0.004** | **1.48(1.13-1.95)** | **0.004** | **0.63** |
| *rs11046182* | *GG* | 554(64.0) | 254(62.9) | 0.144 | 0.704 | 0.95(0.75-1.22) | 0.704 | 0.88(0.68-1.13) | 0.311 | 0.86(0.66-1.12) | 0.265 | - |
|  | *AA+GA* | 312(36.0) | 150(37.1) |  |  | 1.00 |  | 1.00 |  | 1.00 |  |  |
| *rs1799858* | *CC* | 528(61.0) | 272(67.3) | 4.775 | 0.029 | **1.32(1.03-1.69)** | **0.029** | **1.41(1.07-1.85)** | **0.014** | **1.52(1.15-2.01)** | **0.003** | **0.68** |
|  | *TT+CT* | 338(39.0) | 132(32.7) |  |  | 1.00 |  | 1.00 |  | 1.00 |  |  |
| *rs4148671* | *CC* | 744(85.9) | 362(89.6) | 3.339 | 0.068 | 1.00 |  | 1.00 |  | 1.00 |  |  |
|  | *TT+CT* | 122(14.1) | 42(10.4) |  |  | 0.71(0.49-1.03) | 0.069 | 0.73(0.50-1.06) | 0.101 | 0.75(0.51-1.11) | 0.152 | - |
| *rs78148713* | *CC+CT* | 34(3.9) | 24(5.9) | 2.656 | 0.109 | 1.00 |  | 1.00 |  | 1.00 |  |  |
|  | *TT* | 832(96.1) | 380(94.1) |  |  | 0.65(0.38-1.11) | 0.112 | 0.65(0.37-1.13) | 0.125 | 0.62(0.35-1.09) | 0.098 | - |
| *rs145456027* | *CC+CT* | 20(2.3) | 16(4.0) | 2.726 | 0.099 | 1.00 |  | 1.00 |  | 1.00 |  |  |
|  | *TT* | 864(97.7) | 388(96.0) |  |  | 0.57(0.29-1.12) | 0.103 | 0.64(0.32-1.27) | 0.198 | 0.60(0.30-1.23) | 0.162 | - |
| *rs147265929* | *GG+GT* | 72(8.3) | 28(6.9) | 0.727 | 0.394 | 1.00 |  | 1.00 |  | 1.00 |  |  |
|  | *TT* | 794(91.7) | 376(93.1) |  |  | 1.22(0.77-1.92) | 0.395 | 1.50(0.92-2.45) | 0.108 | 1.57(0.95-2.59) | 0.080 | - |
| *rs61928479* | *AA* | 106(12.2) | 32(7.9) | 5.307 | 0.021 | 0.62(0.41-0.93) | 0.022 | 0.68(0.44-1.07) | 0.093 | 0.65(0.41-1.03) | 0.069 | - |
|  | *TT+AT* | 760(87.8) | 372(92.1) |  |  | 1.00 |  | 1.00 |  | 1.00 |  |  |
| *rs141294036* | *CC* | 310(35.8) | 174(43.1) | 6.177 | 0.013 | **1.36(1.07-1.73)** | **0.013** | **1.49(1.07-1.93)** | **0.003** | **1.56(1.19-2.03)** | **0.001** | **0.84** |
|  | *TT+CT* | 556(64.2) | 230(56.9) |  |  | 1.00 |  | 1.00 |  | 1.00 |  |  |

^a^Model 11.

^b^**Model 11e**: It is the same as Model 11, and also including dyslipidemia, such as TRIG, TC, LDL-C, ApoB, HDL-C and ApoA-I.

**Table S19. Association of *KATP* SNPs with increased serum TC level (≥ 4.0 mmol/L) in study participants.**

| ***KATP* SNPs** | | **TC ≥ 4.0 mmol/L (N/%)** | | ***χ2*** | ***P* value** | **Crude**  **OR (95% CI)** | **Crude**  ***P* value** | **Adjusted**  **OR (95% CI)^a^** | **Adjusted**  ***P* value^a^** | **Adjusted**  **OR (95% CI)^b^** | **Adjusted**  ***P* value^b^** |
| --- | --- | --- | --- | --- | --- | --- | --- | --- | --- | --- | --- |
|  |  | **NO** | **YES** |  |  |  |  |  |  |  |  |
| *rs2285676* | *GG* | 188(37.0) | 264(34.6) | 0.742 | 0.389 | 1.00 |  | 1.00 |  | 1.00 |  |
|  | *AA+GA* | 320(63.0) | 498(65.4) |  |  | 1.11(0.88-1.40) | 0.389 | 1.09(0.85-1.40) | 0.506 | 0.85(0.64-1.13) | 0.269 |
| *rs11046182* | *GG* | 324(63.8) | 484(63.5) | 0.009 | 0.924 | 0.99(0.78-1.25) | 0.924 | 0.94(0.73-1.21) | 0.626 | 1.02(0.77-1.36) | 0.883 |
|  | *AA+GA* | 184(36.2) | 278(36.5) |  |  | 1.00 |  | 1.00 |  | 1.00 |  |
| *rs1799858* | *CC* | 326(62.2) | 484(63.5) | 0.225 | 0.635 | 1.06(0.84-1.33) | 0.635 | 1.07(0.84-1.38) | 0.578 | 1.17(0.88-1.56) | 0.272 |
|  | *TT+CT* | 192(37.8) | 278(36.5) |  |  | 1.00 |  | 1.00 |  | 1.00 |  |
| *rs4148671* | *CC* | 448(88.2) | 658(86.4) | 0.915 | 0.339 | 1.00 |  | 1.00 |  | 1.00 |  |
|  | *TT+CT* | 60(11.8) | 104(13.6) |  |  | 1.18(0.84-1.33) | 0.339 | 1.18(0.82-1.70) | 0.382 | 1.37(0.89-2.09) | 0.150 |
| *rs78148713* | *CC+CT* | 30(5.9) | 28(3.7) | 3.481 | 0.062 | 1.00 |  | 1.00 |  | 1.00 |  |
|  | *TT* | 478(94.1) | 734(96.3) |  |  | 1.65(0.97-2.79) | 0.064 | 1.78(0.95-3.34) | 0.072 | 1.55(0.85-2.85) | 0.156 |
| *rs145456027* | *CC+CT* | 14(2.8) | 22(2.9) | 0.019 | 0.890 | 1.00 |  | 1.00 |  | 1.00 |  |
|  | *TT* | 494(97.2) | 740(97.1) |  |  | 0.95(0.48-1.88) | 0.890 | 1.30(0.64-2.63) | 0.475 | 1.38(0.64-2.99) | 0.409 |
| *rs147265929* | *GG+GT* | 36(7.1) | 64(8.4) | 0.724 | 0.395 | 1.00 |  | 1.00 |  | 1.00 |  |
|  | *TT* | 472(92.9) | 698(91.6) |  |  | 0.83(0.54-1.27) | 0.395 | 0.82(0.52-1.29) | 0.383 | 0.93(0.56-1.55) | 0.793 |
| *rs61928479* | *AA* | 54(10.6) | 84(11.0) | 0.049 | 0.825 | 1.04(0.73-1.50) | 0.825 | 1.03(0.69-1.54) | 0.880 | 0.97(0.62-1.52) | 0.892 |
|  | *TT+AT* | 454(89.4) | 678(89.0) |  |  | 1.00 |  | 1.00 |  | 1.00 |  |
| *rs141294036* | *CC* | 200(39.4) | 284(37.3) | 0.570 | 0.450 | 0.92(0.73-1.15) | 0.450 | 0.85(0.66-1.09) | 0.204 | 0.78(0.59-1.04) | 0.089 |
|  | *TT+CT* | 308(60.6) | 478(62.7) |  |  | 1.00 |  | 1.00 |  | 1.00 |  |

^a^Model 11.

^b^**Model 11f**: It is the same as Model 11, and also including dyslipidemia, such as TRIG, LDL-C, ApoB, HDL-C, ApoA-I and Lp(a).

**Table S20. Association of *KATP* SNPs with decreased serum HDL-C level (< 1.0 mmol/L) in study participants.**

| ***KATP* SNPs** | | **HDL-C < 1.0 mmol/L (N/%)** | | ***χ2*** | ***P* value** | **Crude**  **OR (95% CI)** | **Crude**  ***P* value** | **Adjusted**  **OR (95% CI)^a^** | **Adjusted**  ***P* value^a^** | **Adjusted**  **OR (95% CI)^b^** | **Adjusted**  ***P* value^b^** |
| --- | --- | --- | --- | --- | --- | --- | --- | --- | --- | --- | --- |
|  |  | **NO** | **YES** |  |  |  |  |  |  |  |  |
| *rs2285676* | *GG* | 280(35.7) | 172(35.4) | 0.014 | 0.907 | 1.00 |  | 1.00 |  | 1.00 |  |
|  | *AA+GA* | 504(64.3) | 314(64.6) |  |  | 1.01(0.80-1.29) | 0.907 | 0.96(0.74-1.24) | 0.760 | 0.98(0.73-1.32) | 0.883 |
| *rs11046182* | *GG* | 498(63.5) | 310(63.8) | 0.009 | 0.924 | 1.01(0.80-1.28) | 0.924 | 0.99(0.76-1.28) | 0.916 | 0.99(0.73-1.33) | 0.925 |
|  | *AA+GA* | 286(36.5) | 176(36.2) |  |  | 1.00 |  | 1.00 |  | 1.00 |  |
| *rs1799858* | *CC* | 492(62.8) | 308(63.4) | 0.049 | 0.824 | 1.03(0.81-1.30) | 0.824 | 1.08(0.84-1.41) | 0.545 | 1.11(0.82-1.49) | 0.502 |
|  | *TT+CT* | 292(37.2) | 178(36.6) |  |  | 1.00 |  | 1.00 |  | 1.00 |  |
| *rs4148671* | *CC* | 692(88.3) | 414(85.2) | 2.531 | 0.112 | 1.00 |  | 1.00 |  | 1.00 |  |
|  | *TT+CT* | 92(11.7) | 72(14.8) |  |  | 1.31(0.94-1.82) | 0.112 | 1.29(0.86-1.91) | 0.215 | 1.04(0.66-1.62) | 0.877 |
| *rs78148713* | *CC+CT* | 30(3.8) | 28(5.8) | 2.577 | 0.108 | 1.00 |  | 1.00 |  | 1.00 |  |
|  | *TT* | 754(96.2) | 458(94.2) |  |  | 0.65(0.38-1.10) | 0.111 | 0.59(0.34-1.04) | 0.070 | 0.70(0.36-1.35) | 0.286 |
| *rs145456027* | *CC+CT* | 20(2.6) | 16(3.3) | 0.598 | 0.439 | 1.00 |  | 1.00 |  | 1.00 |  |
|  | *TT* | 764(97.4) | 470(96.7) |  |  | 0.77(0.40-1.50) | 0.440 | 0.52(0.25-1.07) | 0.078 | 0.56(0.26-1.23) | 0.150 |
| *rs147265929* | *GG+GT* | 20(2.6) | 16(3.3) | 0.598 | 0.439 | 1.00 |  | 1.00 |  | 1.00 |  |
|  | *TT* | 764(97.4) | 470(96.7) |  |  | 1.11(0.73-1.70) | 0.627 | 1.01(0.64-1.60) | 0.962 | 1.18(0.70-1.99) | 0.530 |
| *rs61928479* | *AA* | 64(8.2) | 36(7.4) | 0.236 | 0.627 | 1.19(0.83-1.71) | 0.336 | 1.09(0.73-1.63) | 0.663 | 0.85(0.54-1.33) | 0.466 |
|  | *TT+AT* | 720(91.8) | 450(92.6) |  |  | 1.00 |  | 1.00 |  | 1.00 |  |
| *rs141294036* | *CC* | 80(10.2) | 58(11.9) | 0.927 | 0.336 | 1.07(0.85-1.35) | 0.570 | 1.02(0.78-1.31) | 0.911 | 1.32(0.97-1.78) | 0.076 |
|  | *TT+CT* | 704(89.8) | 428(88.1) |  |  | 1.00 |  | 1.00 |  | 1.00 |  |

^a^Model 11.

^b^**Model 11g**: It is the same as Model 11, and also including dyslipidemia, such as TRIG, TC, LDL-C, ApoB, ApoA-I and Lp(a).

**Table S21. Association of *KATP* SNPs with increased serum HsCRP level (≥ 3.0 mg/L) in study participants.**

| ***KATP* SNPs** | | **HsCRP ≥ 3.0 mg/L (N/%)** | | ***χ2*** | ***P* value** | **Crude**  **OR (95% CI)** | **Crude**  ***P* value** | **Adjusted**  **OR (95% CI)^a^** | **Adjusted**  ***P* value^a^** | **Adjusted**  **OR (95% CI)^b^** | **Adjusted**  ***P* value^b^** | **Explained variance (%)^b^** |
| --- | --- | --- | --- | --- | --- | --- | --- | --- | --- | --- | --- | --- |
|  |  | **NO** | **YES** |  |  |  |  |  |  |  |  |  |
| *rs2285676* | *GG* | 184(36.5) | 268(35.0) | 0.307 | 0.580 | 1.00 |  | 1.00 |  | 1.00 |  |  |
|  | *AA+GA* | 320(63.5) | 498(65.0) |  |  | 1.07(0.85-1.35) | 0.580 | **1.38(1.03-1.86)** | **0.033** | **1.42(1.05-1.91)** | **0.023** | **0.41** |
| *rs11046182* | *GG* | 308(61.1) | 500(65.3) | 2.276 | 0.131 | 1.20(0.95-1.51) | 0.132 | 1.03(0.77-1.37) | 0.838 | 1.06(0.80-1.40) | 0.693 | - |
|  | *AA+GA* | 196(38.9) | 266(34.7) |  |  | 1.00 |  | 1.00 |  | 1.00 |  |  |
| *rs1799858* | *CC* | 346(68.7) | 454(59.3) | 11.478 | 0.001 | 1.00 |  | 1.00 |  | 1.00 |  |  |
|  | *TT+CT* | 158(31.3) | 312(40.7) |  |  | **1.51(1.19-1.91)** | **0.001** | **1.42(1.07-1.89)** | **0.015** | **1.47(1.10-1.96)** | **0.009** | **0.54** |
| *rs4148671* | *CC* | 448(88.9) | 658(85.9) | 2.414 | 0.120 | 1.00 |  | 1.00 |  | 1.00 |  |  |
|  | *TT+CT* | 56(11.1) | 108(14.1) |  |  | 1.31(0.93-1.85) | 0.121 | 1.36(0.92-2.02) | 0.128 | 1.35(0.90-2.01) | 0.144 | - |
| *rs78148713* | *CC+CT* | 18(3.6) | 40(5.2) | 1.900 | 0.168 | 1.00 |  | 1.00 |  | 1.00 |  |  |
|  | *TT* | 486(96.4) | 726(94.8) |  |  | 0.67(0.38-1.19) | 0.171 | 0.59(0.32-1.11) | 0.102 | 0.61(0.32-1.15) | 0.126 | - |
| *rs145456027* | *CC+CT* | 14(2.8) | 22(2.9) | 0.010 | 0.921 | 1.00 |  | 1.00 |  | 1.00 |  |  |
|  | *TT* | 490(97.2) | 744(97.1) |  |  | 0.97(0.49-1.91) | 0.921 | 0.62(0.28-1.35) | 0.228 | 0.62(0.28-1.36) | 0.230 | - |
| *rs147265929* | *GG+GT* | 40(7.9) | 60(7.8) | 0.004 | 0.947 | 1.00 |  | 1.00 |  | 1.00 |  |  |
|  | *TT* | 464(92.1) | 706(92.2) |  |  | 1.01(0.67-1.54) | 0.947 | 1.21(0.72-1.99) | 0.478 | 1.17(0.70-1.95) | 0.544 | - |
| *rs61928479* | *AA* | 42(8.3) | 96(12.5) | 5.535 | 0.019 | 1.58(1.08-2.31) | 0.019 | 1.36(0.87-2.13) | 0.173 | 1.30(0.83-2.05) | 0.256 | - |
|  | *TT+AT* | 462(91.7) | 670(87.5) |  |  | 1.00 |  | 1.00 |  | 1.00 |  |  |
| *rs141294036* | *CC* | 176(34.9) | 308(40.2) | 3.604 | 0.058 | 1.25(0.99-1.58) | 0.058 | **1.46(1.09-1.95)** | **0.011** | **1.49(1.11-2.00)** | **0.008** | **0.55** |
|  | *TT+CT* | 328(65.1) | 458(59.8) |  |  | 1.00 |  | 1.00 |  | 1.00 |  |  |

^a^**Model 12**: After adjustment for gender, age, smoking, alcohol consumption, WBC, BMI, T2D, SBP, DBP, blood glucose levels (FBS, P2hBS and HbA1C), liver function (ALT, AST and Alb), renal function (Scr, BUN and UA), serum sodium and potassium levels, HsCRP, and dyslipidemia [TRIG, TC, LDL-C, ApoB, HDL-C, ApoA-I and Lp(a)].

^b^**Model 12s**: It is the same as Model 12, and also including RAAS activity (ACE, renin, Ang I, Ang II and ALD).


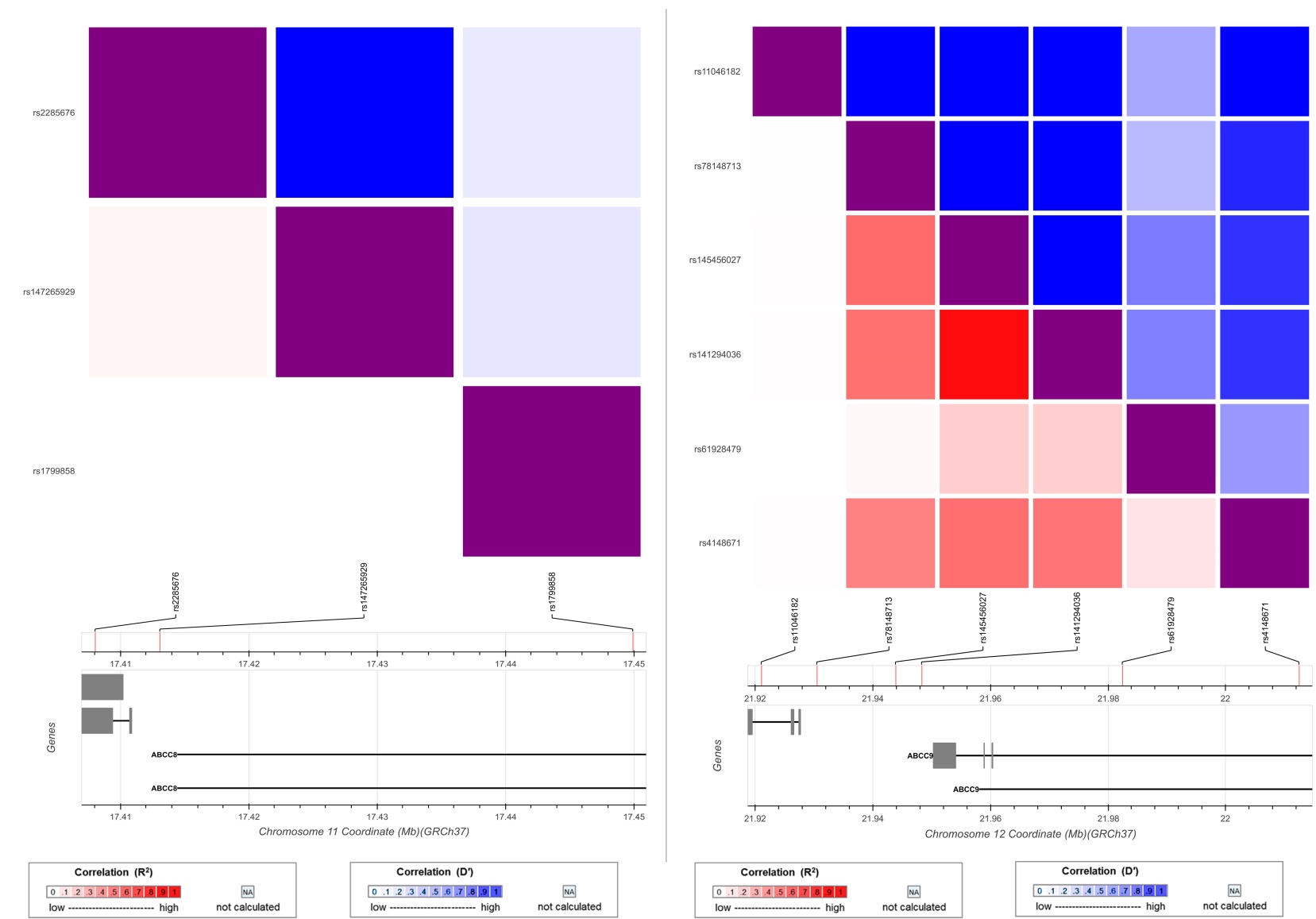


**Figure S1. Schematic representation on the structure, location of polymorphic sites and LD plot of 9 *KATP* SNPs in Chinese (CHS + CHB)^a^.**

**^a^**CHS: Southern Han Chinese, CHB: Han Chinese in Beijing, China.


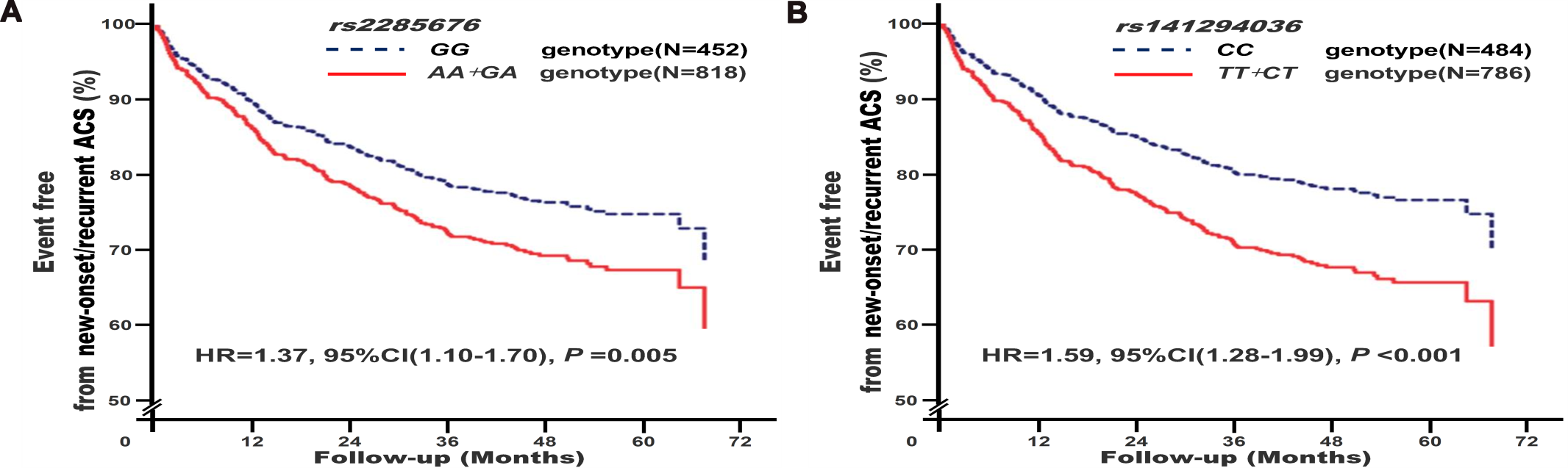


**Figure S2. Association of KATP SNPs with new-onset/recurrent ACS in study participants^a^.**

^a^**Model 2s**: After adjustment for gender, age, smoking, alcohol consumption, BMI, WBC, blood glucose levels (FBS, P2hBS and HbA1C), liver function (ALT, AST and Alb), renal function (Scr, BUN and UA), serum sodium and potassium levels, HsCRP, HbA1C, RAAS activity (ACE, renin, Ang I, Ang II and ALD), dyslipidemia [TRIG, TC, LDL-C, ApoB, HDL-C, ApoA-I and Lp(a)], medical condition (T2D, EH, HF and AF), NYHA functional classification, and echocardiography index (RVD, RAD, LVD, LAD, LVMI, and LVEF),and combined medication (antiplatelet drugs, warfarin, statins, RSIs, BBs, MRA, CCBs, diuretics, digoxin, nitrates, and hypoglycemic agents).


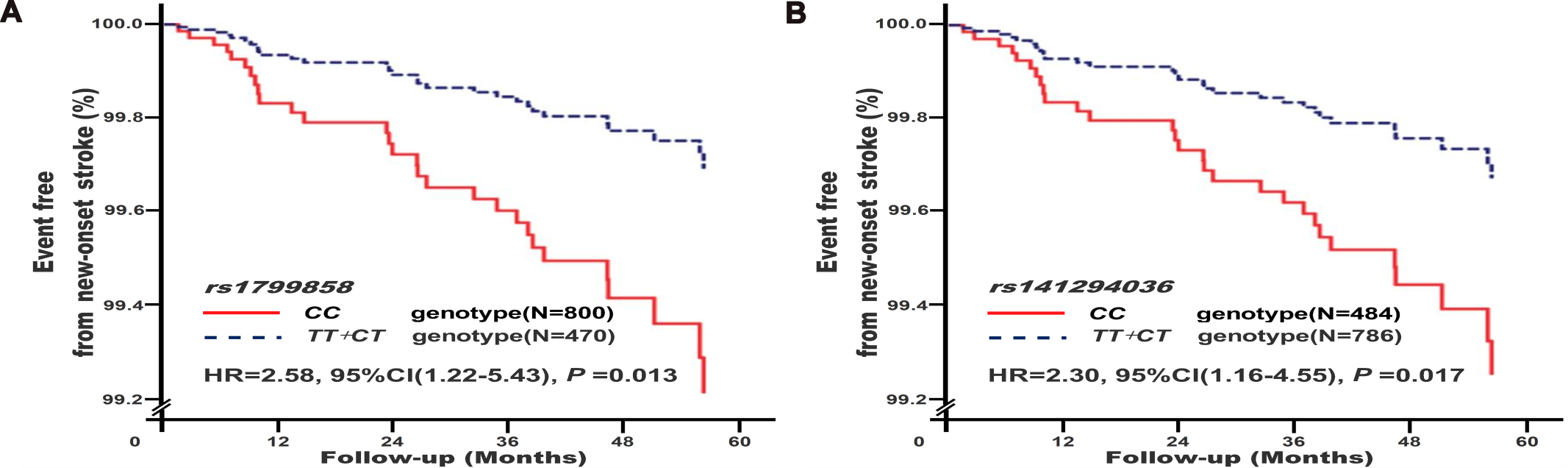


**Figure S3. Association of KATP SNPs with new-onset stroke in study participants^a^.**

^a^**Model 3s**: After adjustment for gender, age, smoking, alcohol consumption, BMI, WBC, blood glucose levels (FBS, P2hBS and HbA1C), liver function (ALT, AST and Alb), renal function (Scr, BUN and UA), serum sodium and potassium levels, HsCRP, RAAS activity (ACE, renin, Ang I, Ang II and ALD), dyslipidemia [TRIG, TC, LDL-C, ApoB, HDL-C, ApoA-I and Lp(a)], medical condition (T2D, EH, CAD(ACS), AF, and LCI), NYHA functional classification, and echocardiography index (RVD, RAD, LVD, LAD, and LVEF), and combined medication (antiplatelet drugs, warfarin, statins, RSIs, BBs, MRA, CCBs, diuretics, digoxin, nitrates, and hypoglycemic agents).


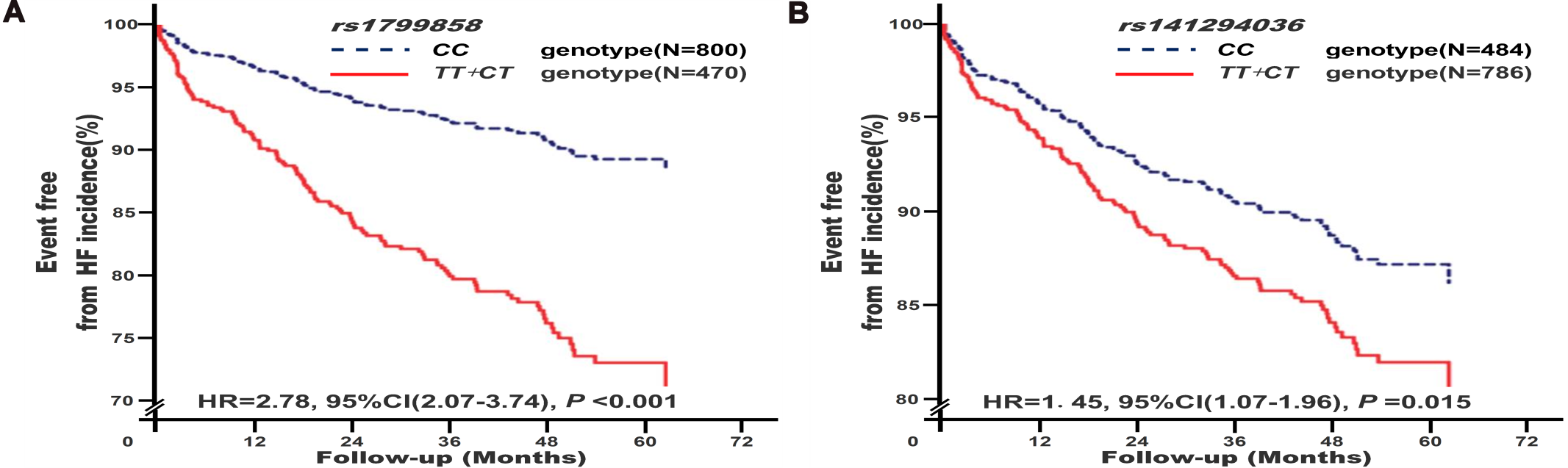


**Figure S4. Association of KATP SNPs with incident HF in study participants^a^.**

^a^**Model 5s**: After adjustment for gender, age, smoking, alcohol consumption, BMI, WBC, blood glucose levels (FBS, P2hBS and HbA1C), liver function (ALT, AST and Alb), renal function (Scr, BUN and UA), serum sodium and potassium levels, HsCRP, RAAS activity (ACE, renin, Ang I, Ang II and ALD), dyslipidemia [TRIG, TC, LDL-C, ApoB, HDL-C, ApoA-I and Lp(a)], medical condition (T2D, HTN, CAD(ACS), and AF), echocardiography index (RVD, RAD, LVD, LAD, and LVEF), and combined medication (antiplatelet drugs, warfarin, statins, RSIs, BBs, MRA, CCBs, diuretics, digoxin, nitrates, and hypoglycemic agents).


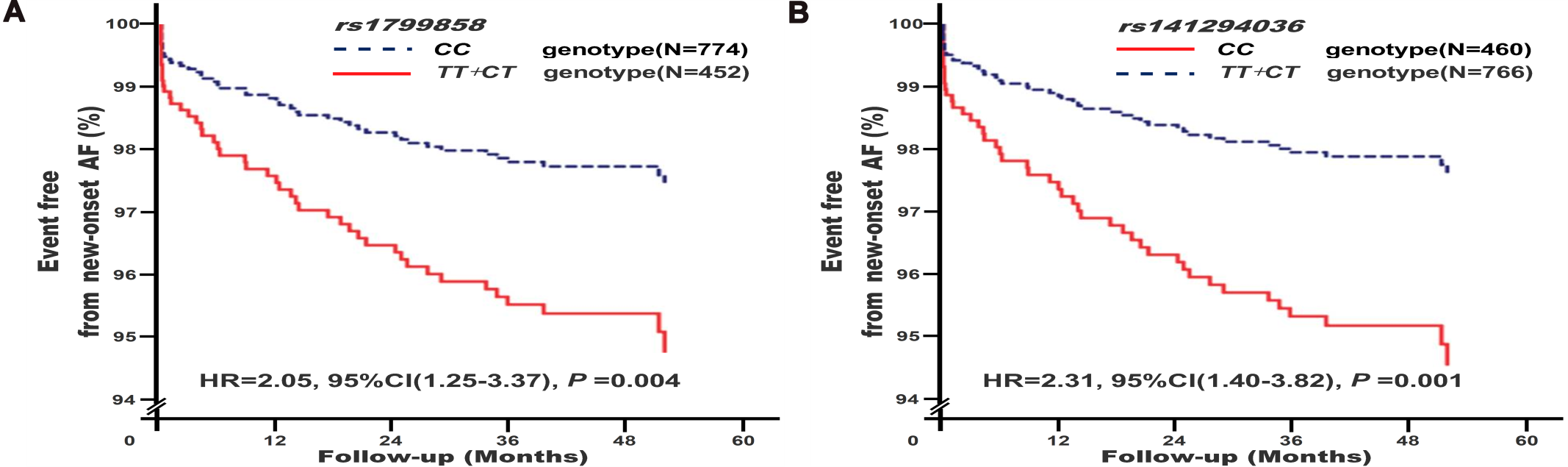


**Figure S5. Association of KATP SNPs with new-onset AF in study participants^a^.**

^a^**Model 8s**: After adjustment for gender, age, smoking, alcohol consumption, BMI, WBC, blood glucose levels (FBS, P2hBS and HbA1C), liver function (ALT, AST and Alb), renal function (Scr, BUN and UA), serum sodium and potassium levels, HsCRP, RAAS activity (ACE, renin, Ang I, Ang II and ALD), dyslipidemia [TRIG, TC, LDL-C, ApoB, HDL-C, ApoA-I and Lp(a)], medical condition (T2D, HTN, CAD(ACS), HF), echocardiography index (RVD, RAD, LVD, LAD, and LVEF), combined medication (antiplatelet drugs, warfarin, statins, RSIs, BBs, MRA, CCBs, diuretics, digoxin, nitrates, and hypoglycemic agents).

**References**

De Jong, G., Kessels, F., and Lodder, J. (2002). Two types of lacunar infarcts: further arguments from a study on prognosis. *Stroke* 33**,** 2072-2076.

January, C.T., Wann, L.S., Alpert, J.S., Calkins, H., Cigarroa, J.E., Cleveland, J.C., Jr., Conti, J.B., Ellinor, P.T., Ezekowitz, M.D., Field, M.E., Murray, K.T., Sacco, R.L., Stevenson, W.G., Tchou, P.J., Tracy, C.M., Yancy, C.W., and Members, A.a.T.F. (2014). 2014 AHA/ACC/HRS guideline for the management of patients with atrial fibrillation: executive summary: a report of the American College of Cardiology/American Heart Association Task Force on practice guidelines and the Heart Rhythm Society. *Circulation* 130**,** 2071-2104.

Lang, R.M., Badano, L.P., Mor-Avi, V., Afilalo, J., Armstrong, A., Ernande, L., Flachskampf, F.A., Foster, E., Goldstein, S.A., Kuznetsova, T., Lancellotti, P., Muraru, D., Picard, M.H., Rietzschel, E.R., Rudski, L., Spencer, K.T., Tsang, W., and Voigt, J.U. (2015). Recommendations for cardiac chamber quantification by echocardiography in adults: an update from the American Society of Echocardiography and the European Association of Cardiovascular Imaging. *Eur Heart J Cardiovasc Imaging* 16**,** 233-270.

Mach, F., Baigent, C., Catapano, A.L., Koskinas, K.C., Casula, M., Badimon, L., Chapman, M.J., De Backer, G.G., Delgado, V., Ference, B.A., Graham, I.M., Halliday, A., Landmesser, U., Mihaylova, B., Pedersen, T.R., Riccardi, G., Richter, D.J., Sabatine, M.S., Taskinen, M.R., Tokgozoglu, L., Wiklund, O., and Group, E.S.C.S.D. (2020). 2019 ESC/EAS Guidelines for the management of dyslipidaemias: lipid modification to reduce cardiovascular risk. *Eur Heart J* 41**,** 111-188.

Wardlaw, J.M., Smith, E.E., Biessels, G.J., Cordonnier, C., Fazekas, F., Frayne, R., Lindley, R.I., O'brien, J.T., Barkhof, F., Benavente, O.R., Black, S.E., Brayne, C., Breteler, M., Chabriat, H., Decarli, C., De Leeuw, F.E., Doubal, F., Duering, M., Fox, N.C., Greenberg, S., Hachinski, V., Kilimann, I., Mok, V., Oostenbrugge, R., Pantoni, L., Speck, O., Stephan, B.C., Teipel, S., Viswanathan, A., Werring, D., Chen, C., Smith, C., Van Buchem, M., Norrving, B., Gorelick, P.B., Dichgans, M., and Neuroimaging, S.T.F.R.V.C.O. (2013). Neuroimaging standards for research into small vessel disease and its contribution to ageing and neurodegeneration. *Lancet Neurol* 12**,** 822-838.
